# Relationship between histone modifications and transcription factor binding is protein family specific

**DOI:** 10.1101/gr.220079.116

**Published:** 2018-03

**Authors:** Beibei Xin, Remo Rohs

**Affiliations:** Computational Biology and Bioinformatics Program, Departments of Biological Sciences, Chemistry, Physics & Astronomy, and Computer Science, University of Southern California, Los Angeles, California 90089, USA

## Abstract

The very small fraction of putative binding sites (BSs) that are occupied by transcription factors (TFs) in vivo can be highly variable across different cell types. This observation has been partly attributed to changes in chromatin accessibility and histone modification (HM) patterns surrounding BSs. Previous studies focusing on BSs within DNA regulatory regions found correlations between HM patterns and TF binding specificities. However, a mechanistic understanding of TF–DNA binding specificity determinants is still not available. The ability to predict in vivo TF binding on a genome-wide scale requires the identification of features that determine TF binding based on evolutionary relationships of DNA binding proteins. To reveal protein family–dependent mechanisms of TF binding, we conducted comprehensive comparisons of HM patterns surrounding BSs and non-BSs with exactly matched core motifs for TFs in three cell lines: 33 TFs in GM12878, 37 TFs in K562, and 18 TFs in H1-hESC. These TFs displayed protein family–specific preferences for HM patterns surrounding BSs, with high agreement among cell lines. Moreover, compared to models based on DNA sequence and shape at flanking regions of BSs, HM-augmented quantitative machine-learning methods resulted in increased performance in a TF family–specific manner. Analysis of the relative importance of features in these models indicated that TFs, displaying larger HM pattern differences between BSs and non-BSs, bound DNA in an HM-specific manner on a protein family–specific basis. We propose that TF family–specific HM preferences reveal distinct mechanisms that assist in guiding TFs to their cognate BSs by altering chromatin structure and accessibility.

Unraveling the mechanisms of how transcription factors (TFs) achieve DNA binding specificities in vivo is vital for understanding transcriptional regulation. The relatively short core-binding motifs of TFs can appear numerous times in a genome, but only a very small fraction of these putative binding sites is functional (Landolin et al. 2010; Spitz and Furlong 2012). TFs can precisely identify their functional binding sites from among the other 99.8% of putative binding sites in a cellular environment in vivo (Wang et al. 2012)*.* Given the multiple layers that contribute to in vivo binding (Levo and Segal 2014; Slattery et al. 2014; Zentner et al. 2015; Mathelier et al. 2016b), it is clear that DNA sequence and shape at core binding sites, which in vitro experiments have identified as determinants of DNA binding specificity (Zhao and Stormo 2011; Gordân et al. 2013; Abe et al. 2015; Levo et al. 2015; Zhou et al. 2015; Yang et al. 2017), are not sufficient to explain TF binding in vivo*.* An important question is how TFs distinguish their functional binding sites (BSs) in one region of the genome from putative non-BSs with exactly matched core motifs in other regions in vivo. Multiple factors that may explain this behavior include chromatin accessibility, cooperativity, epigenetic marks, and sequence context (Slattery et al. 2014; Dror et al. 2016; Inukai et al. 2017). Among these factors, chromatin inaccessibility can largely explain non-BSs because motifs occupied by histones are generally not accessible to TFs (Song et al. 2011). Base pairs in flanking regions of core BSs can affect TF binding through their effects on local DNA structure (Rohs et al. 2010; Levo and Segal 2014). Nucleosome occupancy exerts additional influence on TF binding (Kornberg and Lorch 1999; Pique-Regi et al. 2011). Epigenetic marks are cell-type–specific signatures (He et al. 2017) that contribute to cell-type–specific protein binding events (Zhu et al. 2013).

Epigenetic studies suggested that posttranscriptional histone modifications (HMs) play a central role in transcriptional regulation and revealed substantial overlaps between TF BSs and HM marks (The ENCODE Project Consortium 2012). Recent work introduced approaches for the quantitative modeling of relationships between TF binding and HM patterns (Benveniste et al. 2014; Liu et al. 2015). Despite the reported relationship between TF binding and HM patterns, mechanisms that cause this relationship are still unknown. Moreover, it is unclear whether this relationship varies between TF families, or if it can reveal mechanisms of TF binding on a protein family–specific basis. HMs are small changes at nucleosome surfaces that can significantly affect the chromatin tertiary structure and compaction (Lu et al. 2008; Glatt et al. 2011). From a structural perspective, one may ask whether HM patterns are conserved around the in vivo BSs of TFs and whether this relationship varies among protein families. However, genomic data will need to be analyzed to answer these questions, given the paucity of structural information about proteins bound to nucleosomes with HM marks. Although HM patterns in the BS environment are known to contribute to TF binding, this relationship is not yet understood from a mechanistic perspective.

In this work, we performed a large-scale analysis of how HM patterns contribute to TF binding specificities for many protein families. We asked whether certain TFs or TF families exhibit different HM patterns between BSs and non-BSs compared to other TFs or TF families. To answer this question, we built machine-learning models to distinguish BSs and non-BSs by using combinations of DNA sequence and shape features at flanking regions and HM patterns surrounding DNA binding motifs. Based on the extent to which these different features contribute to TF binding specificities, we discuss whether TF families utilize different binding mechanisms at regions extending beyond their core motifs. Our work represents a step toward a better understanding of the relationship between histone modifications and TF binding.

## Results

### HM patterns surrounding TF BSs and non-BSs show conserved patterns in vivo

To study the different HM patterns surrounding TF BSs and non-BSs and the conservation of these patterns, we downloaded data from the ENCODE Consortium (Supplemental Table S1; http://genome.ucsc.edu/ENCODE/downloads.html) for three human cell lines: B-lymphoblastoid cells (GM12878), erythrocytic leukemia cells (K562), and embryonic stem cells (H1-hESC). We collected genome-wide TF binding profiles for 44 TFs in GM12878, 43 TFs in K562, and 24 TFs in H1-hESC cells, and considered 10 HMs at histone tails (H3K4me2, H3K27ac, H3K4me1, H3K4me3, H3K79me2, H3K9ac, H3K9me3, H4K20me1, H3K27me3, and H3K36me3). These TF binding profiles and HM profiles were generated by chromatin immunoprecipitation combined with sequencing (ChIP-seq) assays (Bernstein et al. 2005).

**Figure 1.**
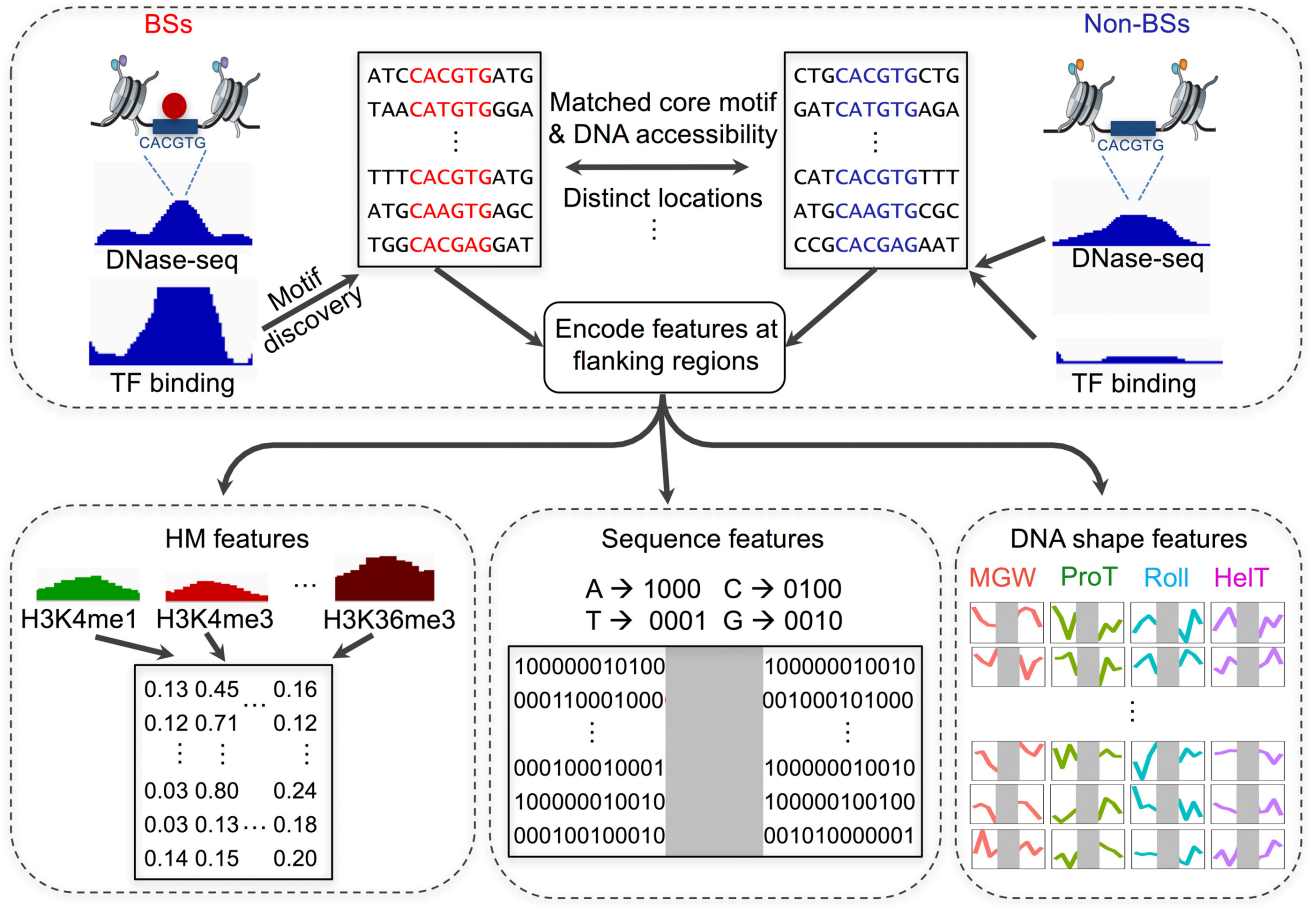
Flowchart describing the approach of modeling BSs and non-BSs with DNA sequence, DNA shape features at flanking regions, and HM features. In each cell line, chromatin-accessible regions derived from DNase-seq data were genomic regions of interest. For each TF, sequences at ChIP-seq peaks were first aligned using position frequency matrices (PFMs) to obtain BSs. For each BS, an exactly matched motif was found from chromatin-accessible and distinct genomic regions as a motif pool. Within the motif pool, motif sets with similar chromatin accessibility distributions as the BSs were selected as non-BSs. With a set of BSs and non-BSs, DNA sequence and four DNA shape features, as well as 10 HM patterns, were calculated for flanking regions and fed to downstream modeling to distinguish BSs and non-BSs.

To make reasonable comparisons of HM patterns around TF BSs and non-BSs, our experiments focused on BSs and non-BSs selected from regions that had similar levels of chromatin accessibility and exactly matched core motifs (Methods). We performed motif discovery on ChIP-seq peaks by using FIMO (Grant et al. 2011), obtaining a set of BSs for each TF, and selected non-BSs with the following assumptions. First, BSs and non-BSs in the human genome were assumed to be located in regions with different levels of chromatin accessibility (Wang et al. 2012). To exclude this effect for a valid control, non-BSs were selected to have distributions of chromatin accessibility that were similar to those of the BSs of a given TF (Supplemental Fig. S1). Chromatin-accessible regions were obtained with the DNase-seq technique (Hesselberth et al. 2009; Boyle et al. 2011). Second, to avoid the effect of primary sequence preference at core motifs, non-BSs were chosen based on their having exactly matched core motifs with the BSs. Third, selected non-BSs were located at distinct genomic locations and had sample sizes that were similar to those of the BSs for modeling consistency. A flowchart describing the analysis is shown in Figure 1. This experimental setup for defining BSs and non-BSs enabled us to focus directly on HM pattern differences between BSs and non-BSs.

**Figure 2.**
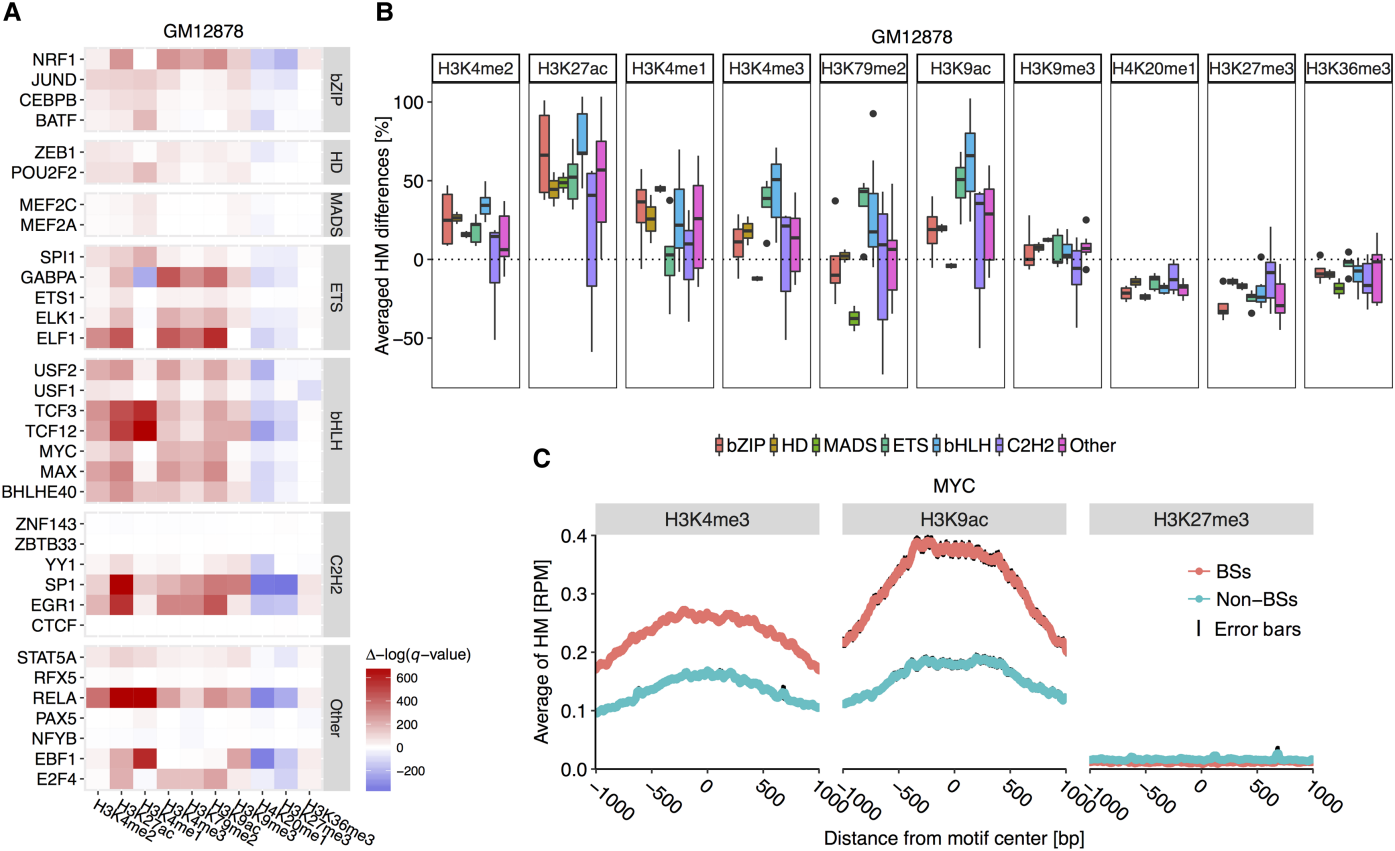
TF families show conserved differences in HM patterns between BSs and non-BSs. (*A*) Heat map displaying results of statistical comparison between HM levels at positions 1 kb upstream of and downstream from BSs and non-BSs in the GM12878 cell line. Positive Δ[−log(*q*-value)], in red, indicates BS environments with significantly higher HM levels compared to non-BS environments. Negative Δ[−log(*q*-value)], in blue, indicates BS environments with lower HM levels. The MADS-domain, C2H2, ETS, and bHLH TF families show conserved HM pattern differences. (*B*) Average HM differences across TF families in the GM12878 cell line. Centerlines of box plots represent medians, edges indicate the first and third quartiles, and whiskers indicate minimum/maximum values within 1.5 times the interquartile from the box. This setup for displaying box plots is consistent in subsequent box plots. (*C*) Average H3K4me3, H3K9ac, and H3K27me3 levels at each position 1 kb upstream of and downstream from BSs and non-BSs for MYC (bHLH family). Black edges encompassing the average line represent standard error bars at each nucleotide position.

We removed TFs that had fewer than 132 genomic binding locations or lacked binding motifs at the ChIP-seq peaks so that they could be aligned (Methods; Supplemental Methods). As a result, 33, 37, and 18 TFs remained for further analysis in the GM12878, K562, and H1-hESC cell lines, respectively. With these data, we were first interested in the HM pattern differences around aligned BSs and non-BSs for each TF in the GM12878 cell line. We examined HM patterns at single-base-pair resolution within regions of 1 kb upstream of and downstream from the BSs and non-BSs, and then calculated the average HM patterns. For these considered TFs, the average HM levels of H3K27me3, H3K36me3, and H4K20me1 were reduced by 21%, 9.6%, and 17%, respectively, in the environment of BSs compared to non-BSs. Average HM levels of H3K4me2, H3K27ac, H3K4me3, H3K79me2, and H3K9ac were elevated by 18%, 52%, 19%, 8%, and 31%, respectively, for regions containing BSs compared to non-BSs (Fig. 2B). These substantial differences in HM patterns for TFs could not be detected when we randomly shuffled the labels between BSs and non-BSs.

We evaluated the statistical significance of HM pattern differences between BSs and non-BSs (Methods) and clustered TFs based on Pfam binding domains (Finn et al. 2014). Evolutionarily related TFs displayed similarly substantial HM pattern differences. Specifically, TFs from the ETS (three of five) and bHLH (seven of seven) families showed larger HM pattern differences between BSs and non-BSs, whereas TFs from the bZIP (three of four), homeodomain (HD; two of two), and C2H2 (four of six) families displayed smaller HM pattern differences in the GM12878 cell line (Fig. 2A). This observation indicates that TFs with evolutionarily related DNA binding domains sample putative BSs with similar HM pattern environments.

To investigate the conservation of HM pattern preferences for TFs from various protein families, we applied a similar analysis to the K562 and H1-hESC cell lines. We found that HM preferences were conserved for the MADS-domain, ETS, bHLH, bZIP, and C2H2 families (Supplemental Figs. S2, S3). In addition, the GATA and STAT families displayed HM pattern differences that were similar to preferences of the bHLH family in the K562 cell line. Within TF families, the bZIP and C2H2 families showed more diverse HM pattern differences than the MADS-domain, ETS, and bHLH families (Fig. 2A; Supplemental Figs. S2, S3). This observation is in agreement with the fact that some TFs from the C2H2 family, despite having conserved secondary structures of zinc fingers and linkers, still have dynamic linker structures and diverse conformations in their unbound state prior to DNA binding. These TFs also have diverse position weight matrices (Supplemental Fig. S4) and require binding to correct DNA sequences to adopt a stable protein structure (Laity et al. 2001). In addition to having larger diversity in the core binding site sequence of C2H2 zinc fingers, their BSs show more differences in binding energy, GC content, and DNA shape profiles than do other protein families (Supplemental Figs. S5, S6; Supplemental Table S3).

These protein family–specific and cell-type–consistent HM patterns in the environment of in vivo BSs seemed to reveal particular differences when only small percentages of BSs overlapped among different cell lines. For TFs appearing in the GM12878 cell line, the average percentage of overlapping BSs between any two cell lines was 30% (ranging from 2%–84%), and the overlap among the three cell lines was 19% (ranging from 0.4%–70%) (Supplemental Table S2). These observations indicate that fewer than half of the BSs were shared among different cell lines. We further analyzed MYC BSs in the GM12878 and K562 cell lines and partitioned the entire set of BSs into three subsets: Group 1 included 852 BSs in the GM12878 but not the K562 cell line; group 2 included 310 BSs shared in both cell lines; and group 3 included 4872 BSs in the K562 but not the GM12878 cell line (Supplemental Fig. S7).

Because high H3 K4/K79 methylation and H3 acetylation levels are prerequisites for MYC binding in vivo (Guccione et al. 2006), we examined the distribution of H3K4me3, H3K79me2, and H3K9ac patterns among these three groups of BSs. Two of the three HM marks surrounding group 1 BSs had high levels in the GM12878 cell line and low levels in the K562 cell line (one-sided paired *t*-test *P*-values: 6.2 × 10^−7^ for H3K4me3 and 1.6 × 10^−20^ for H3K9ac). In contrast, these HM levels surrounding group 3 BSs were higher in the K562 than in the GM12878 cell line (one-sided paired *t*-test *P*-values: 1.1 × 10^−10^ for H3K4me3, 1.2 × 10^−38^ for H3K79me2, and 3.9 × 10^−7^ for H3K9ac). Despite poor conservation of the BSs in terms of their genomic location, most of the considered TFs displayed conserved HM patterns among different cell lines.

To exemplify how HM patterns are distributed in motif environments of TF BSs, we displayed average HM patterns of H3K4me3, H3K9ac, and H3K27me3 at each position 1 kb upstream of and downstream from BSs and non-BSs for MYC, a TF from the bHLH family, in the GM12878 cell line (Fig. 2C). H3K4me3 and H3K9ac are crucial for in vivo MYC binding. Consistent with this fact, the average H3K4me3 and H3K9ac levels in the motif environment of BSs were higher compared to non-BS regions. As a control, average H3K27me3 levels were similar in environments of BSs and non-BSs, and our observations in the K562 and H1-hESC cell lines were consistent (Supplemental Figs. S2, S3). To compare HM patterns for TF families with fewer differences between BSs and non-BSs (Fig. 2A), we plotted average HM patterns for H3K4me3, H3K9ac, and H3K27me3 for CEBPB from the bZIP family (Supplemental Fig. S8). As we had expected, these three HMs showed similar patterns surrounding BSs and non-BSs of CEBPB.

Upon binding DNA in closed chromatin regions (irrespective of the presence of nucleosomes), pioneer factors recruit chromatin remodelers and histone-modifying enzymes, disrupt the chromatin structure, and reprogram epigenetic marks (Magnani et al. 2011; Zaret and Carroll 2011; Vernimmen and Bickmore 2015). The TFs considered in this study included pioneer factors from different TF families, including GATA2 from the GATA family (Anguita et al. 2002), NFYB (Oldfield et al. 2014) from the NFY family, SPI1 from the ETS family (Wang et al. 2014), and RFX5 from the family of RFX-related factors (Gauthier et al. 2012). Moreover, pioneer factors colocalize with other TFs in a cell-line–specific manner. For example, NFY extensively colocalizes only with USF1 and FOS at inactive chromatin domains in the K562 cell line (Fleming et al. 2013). We observed that these pioneer factors, except for those from the GATA family, and colocalized TFs showed similar HM patterns in environments of BSs compared to non-BSs (Fig. 2; Supplemental Figs. S2, S3).

### HM patterns in the BS environment contribute to quantitative predictions of in vivo TF binding

Our qualitative analysis of HM patterns between BSs and non-BSs of TFs revealed similar and conserved differences for various protein families. Therefore, we were interested in whether the HM patterns contribute quantitatively to the discrimination of BSs versus non-BSs for TFs and whether those contributions are also protein family–dependent. We previously showed that DNA sequence information and four DNA shape features (i.e., minor groove width [MGW], propeller twist [ProT], Roll, and helix twist [HelT]) at flanking regions contribute to the quantitative modeling of TF binding specificities both in vitro and in vivo (Gordân et al. 2013; Dror et al. 2015). Therefore, we built L2-regularized multiple linear regression (MLR) models, incorporating various combinations of DNA sequence and shape features at 10-bp flanking regions and 10 average HM patterns in an environment of 1 kb upstream of and downstream from motifs, to classify previously defined BSs and non-BSs (Methods).

BSs and non-BSs were described as feature vectors containing distinct sets of features (i.e., DNA sequence and shape at nucleotide resolution, and average HM levels). DNA sequence features are binary categorical attributes characterizing the chemical identity of base pairs. This information encodes hydrogen bonds and other direct contacts between amino acids and base pairs in predominantly the major groove (Rohs et al. 2010). DNA shape features are contiguous attributes capturing DNA shape properties and electrostatic interactions predominantly in the minor groove (Rohs et al. 2009; Chiu et al. 2017). HM levels are contiguous attributes that describe surrounding epigenetic marks that may be sensitive to TF binding (Grubert et al. 2015) and can also be primed for the binding of specific TFs (Ziller et al. 2015). These three types of feature categories represent different mechanisms of in vivo TF binding specificities.

After collecting binding data and encoding features for each BS sequence, we implemented two different models: sequence+shape models, using a combination of DNA sequence and shape features; and sequence+shape+HM models, using a combination of DNA sequence, DNA shape, and HM pattern features. To examine how these models perform quantitatively as a function of the lengths of flanking regions used in calculating HM patterns, we tried different length scales ranging from 10 to 2000 bp. Similarly, we tried flanking region lengths of 5, 10, and 15 bp for calculating DNA sequence and shape features. We used the area under the precision and recall curve (AUPRC) to evaluate the performance of the models.

**Figure 3.**
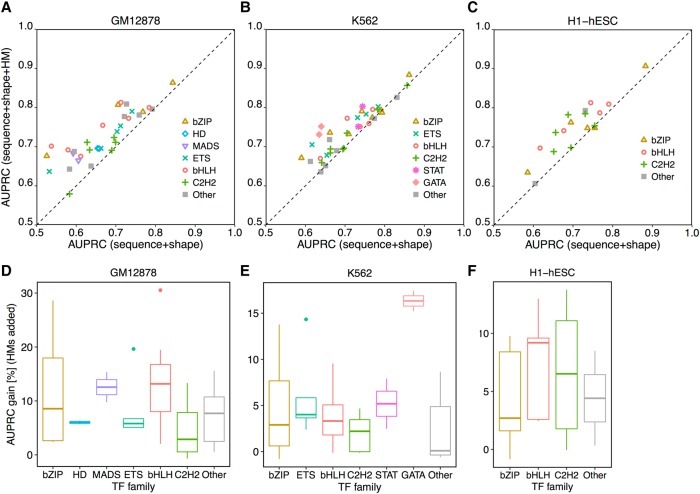
HM patterns of the BS environment largely contribute to the quantitative prediction of in vivo TF binding in a protein family–specific manner. L2-regularized MLR models were implemented to distinguish BSs and non-BSs for TFs from different families. AUPRC was used to measure performance of different models. Comparisons of models are shown between sequence+shape and sequence+shape+HM features in the GM12878 (*A*), K562 (*B*), and H1-hESC (*C*) cell lines. Extents of performance gain in HM-augmented models are protein family specific in the GM12878 (*D*), K562 (*E*), and H1-hESC (*F*) cell lines.

Sequence+shape+HM models achieved average AUPRCs of 0.73, 0.74, and 0.75 for TFs considered in the GM12878, K562, and H1-hESC cell lines (Supplemental Table S4). Adding HM patterns increased the performance of discriminating BSs from non-BSs (Fig. 3A–C). For example, because certain HM patterns are prerequisites for MYC binding, adding HM patterns to sequence+shape models yielded a 14.0% increase in AUPRC (from 0.71 to 0.81) in the GM12878 cell line. Moreover, performances of sequence+shape+HM models did not show strong length-scale dependencies in calculating HM patterns (Supplemental Figs. S9–S11) or in calculating DNA sequence and shape features (Supplemental Figs. S12–S14).

The extent to which the inclusion of HM patterns in the models improved the prediction accuracy of TF binding specificities was protein family specific. With consistent and substantially different HM patterns (Fig. 2A), TFs from the bHLH family had median performance boosts of 13.2%, 3.3%, and 9.2% when using HM-augmented models in the three cell lines. In contrast, TFs from the C2H2 family benefited comparatively less, with median performance improvements of 2.9%, 2.2%, and 6.5% in the three cell lines. Moreover, the performance improvements were distributed over a wider range (Fig. 3D–F; Supplemental Fig. S15). TFs from the MADS-domain family in the GM12878 cell line and the GATA family in the K562 cell line also showed a substantial performance boost when HM-augmented models were used. Using imbalanced data did not change these observations (Methods; Supplemental Figs. S16, S17).

### TF families vary in their preferences for DNA sequence and shape features and HM patterns

Determinants affecting in vivo TF binding are highly correlated and have not yet been deconvolved. For example, the GC content of a BS region can affect nucleosome positioning (Brogaard et al. 2012). DNA shape features are derived from nucleotide sequences (Zhou et al. 2013), which are closely related to HM patterns (Henikoff and Shilatifard 2011; Benveniste et al. 2014; Grubert et al. 2015). With HM-augmented models that can increase the modeling accuracy of DNA binding specificities across TF families, we further separated the contributions from DNA sequence and shape at flanking regions and the contribution from HM patterns.

**Figure 4.**
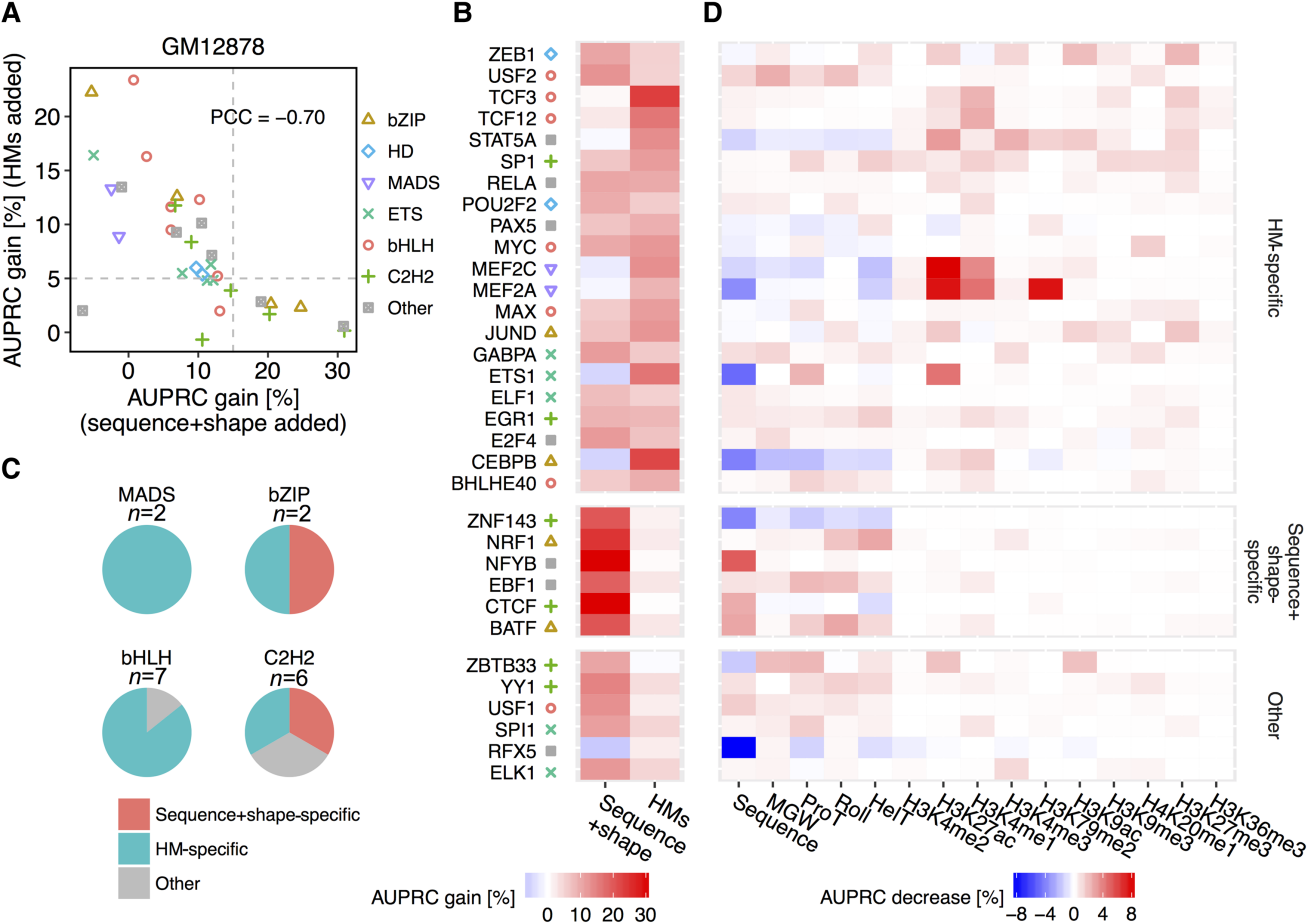
Deconvolution of DNA sequence and shape features at flanking regions and 10 HM patterns in the GM12878 cell line. (*A*) Scatter plot showing performance gain through adding different sets of features. The *x*-axis represents HM pattern-only models as baseline, and recorded performance increases through adding DNA sequence and shape features at flanking regions. The *y*-axis represents models based on DNA sequence and shape features at flanking regions as baseline, and recorded performance increases through adding HM pattern features. Gray dashed lines intersect with *x*-axis at 15% and with *y*-axis at 5%. The Pearson correlation coefficient (PCC) was calculated between AUPRC gain through adding these two sets of features. (*B*) Heat map displaying performance gains when adding either sequence+shape features or HM patterns. With cutoffs as shown by the gray dashed line in *A*, TFs were grouped into sequence+shape specific, HM specific, and a group with other features preferred. (*C*) Pie charts showing the number of TFs with different binding mechanisms in the MADS-domain, bHLH, bZIP, and C2H2 TF families. (*D*) Heat map representing the percentage decrease of AUPRC in leave-one-feature-out experiments compared to complete models considering DNA sequence and shape features, and 10 HM features. A more intense red color in a cell indicates a greater performance decrease when leaving out the feature displayed in the *x*-axis for the TF displayed in the *y*-axis.

Our previous work was aimed at deconvolving determinants of in vitro TF binding (Abe et al. 2015). Here, we applied a similar strategy to investigate the importance of DNA sequence and shape features at flanking regions that could not be explained by HM features, or vice versa. Specifically, we evaluated the importance of individual sequence+shape or HM features by using performance increases relative to sequence+shape, HM-only, or sequence+shape+HM models. For most of the TFs considered, contributions of sequence+shape and HM features had a strong negative relationship (Fig. 4A), indicating a more general phenomenon that the degree to which flanking regions contribute to TF binding can be attenuated by the chromatin context (Levo and Segal 2014). In general, if DNA flanking regions are more likely occupied by nucleosomes, then the HM patterns contribute more than DNA sequence and shape features to TF binding specificity predictions.

We further observed that TFs from the GATA and MADS-domain families were distributed in the upper left quadrant of the plot in Figure 4A, with HM patterns showing larger contributions than DNA sequence and shape features to TF binding specificity predictions. On the other hand, TFs from the bZIP and C2H2 families were more broadly distributed in the scatter plot. When we selected an AUPRC increase of 5% or 15% upon adding HMs and sequence+shape features (Fig. 4A, gray dashed lines), respectively, to be the “feature importance” threshold (i.e., for a set of features to be considered important for TF binding), the TFs separated into three groups. The TFs in the upper left quadrant of the scatter plot in Figure 4A were termed “HM specific,” those in the bottom right quadrant were termed “sequence+shape specific,” and those in the bottom left quadrant were regarded as “other” (Fig. 4B). For example, all but one (USF1) of the five TFs in the bHLH family were observed to bind DNA in an HM-specific manner (Fig. 4C). Comparatively, for TFs in the bZIP and C2H2 families, HM features did not always represent important features. We noticed, however, that binding mechanisms were protein family specific and consistent in the three cell types. Specifically, TFs from the C2H2 and bZIP families were found to bind in both a DNA sequence+shape-specific and an HM-specific manner, whereas most of the TFs from the bHLH, GATA, and MADS-domain families tended to bind in an HM-specific manner (Fig. 4C; Supplemental Figs. S18, S19).

TFs from the bHLH family tended to bind mostly in an HM-specific manner. They exhibited consistent and increased H3K4me3, H3K79me2, and H3K9ac patterns in the motif environment of their in vivo BSs (Fig. 2A). These TFs might require primed HM patterns to achieve DNA binding specificity (Guccione et al. 2006; Ziller et al. 2015). USF1 and USF2, as exceptions, were found to bind in a sequence+shape-specific manner in the K562 cell line. On one hand, when accounting for the preferences of flanking base pairs, in vivo BSs for TFs of the bHLH family showed increased in vitro binding signals (Gordân et al. 2013). Differences in the one or two proximal base pairs directly flanking the E-box at promoter regions appeared to alter the transcriptional rates (Aow et al. 2013; Rajkumar et al. 2013). On the other hand, the lesser importance of HM patterns for USF1 binding can be explained by its frequent cobinding with pioneer factors.

TFs from the GATA family are known as pioneer factors. As such, confirming the binding preference of these TFs might require the analysis of time-resolved HM pattern changes surrounding the BSs. This possibility is because the large HM pattern differences between BSs and non-BSs (Supplemental Fig. S2) might be due to HM pattern changes upon TF binding (Magnani et al. 2011; Zaret and Mango 2016).

TFs from the STAT family used both sequence+shape and HM features at flanking regions to achieve DNA binding specificity. STAT1, for instance, has independently derived BSs that associate strongly with regions of H3K4me1 and H3K4me3 histone marks in HeLa cells (Robertson et al. 2008). Examining three flanking positions upstream and two flanking positions downstream, it was reported that STAT1, STAT5, and STAT6 revealed preferences for certain base pairs in the flanking regions (Ehret et al. 2001). The C2H2 family, which binds DNA using the most promiscuous mechanisms, showed various HM pattern preferences in regions surrounding BSs (Fig. 2A; Supplemental Figs. S2, S3). For TFs exhibiting large differences in HM patterns, HM changes in the motif environment of BSs might be due to initial interactions with histone-modifying enzymes, followed by changes in HM patterns. For instance, YY1 interacts with histone acetyltransferase EP300 (Lee et al. 1995), CREB-binding protein (CBP) (Austen et al. 1997), and histone deacetylase 1 (HDAC1), HDAC2, and HDAC3 (Yang et al. 1996). Such HM pattern changes can be explained by DNA variants that are highly related to alterations in TF binding intensities (Grubert et al. 2015). These TFs may approach their BSs through initially sampling DNA sequence and shape (Dror et al. 2016), followed by causing or stabilizing HM pattern changes around the BSs.

We also investigated the importance of individual features that cannot be explained by other features in predicting binding specificities for each TF. Starting from the HM-augmented model, we individually removed DNA sequence or shape features or one of the 10 HM patterns, built a series of leave-one-feature-out L2-regularized MLR models, and recorded AUPRC decreases to evaluate model performance (Fig. 4D; Supplemental Figs. S18, S19; Methods). For most TFs of the bHLH family, flanking regions around BSs contributed to TF binding specificity more substantially through their local DNA structure than through DNA sequence, as previously reported (Gordân et al. 2013).

Among these DNA shape features, ProT was important for the bHLH family in all three cell lines (Dror et al. 2015). H3K4me2, as an activating mark, was significantly different in regions surrounding BSs of TFs from the bHLH and ETS families. The same histone mark, however, showed lesser importance in the three cell types, implying co-occurrence with other activating marks such as H3K4me3 or H3K27ac (Peach et al. 2012; Du et al. 2013). Comparatively, H3K4me1 carries unique information and distinguishes active from poised enhancers when combined with H3K27ac and H3K27me3, respectively (Wei et al. 2009; Creyghton et al. 2010; Rada-Iglesias et al. 2011). For TFs that exhibit HM patterns as an important feature category, H3K4me1 usually indicates a substantial contribution.

### Preferences for HM patterns can constrain TF co-occupancy

Our data showed that HM pattern differences were degenerate characteristics in defining TF binding specificities (Fig. 2A). An intriguing question was how HM pattern preferences of TFs correlate with the cobinding of TFs, given previous observations that TFs tend to bind DNA in a cooperative manner (Mann et al. 2009).

**Figure 5.**
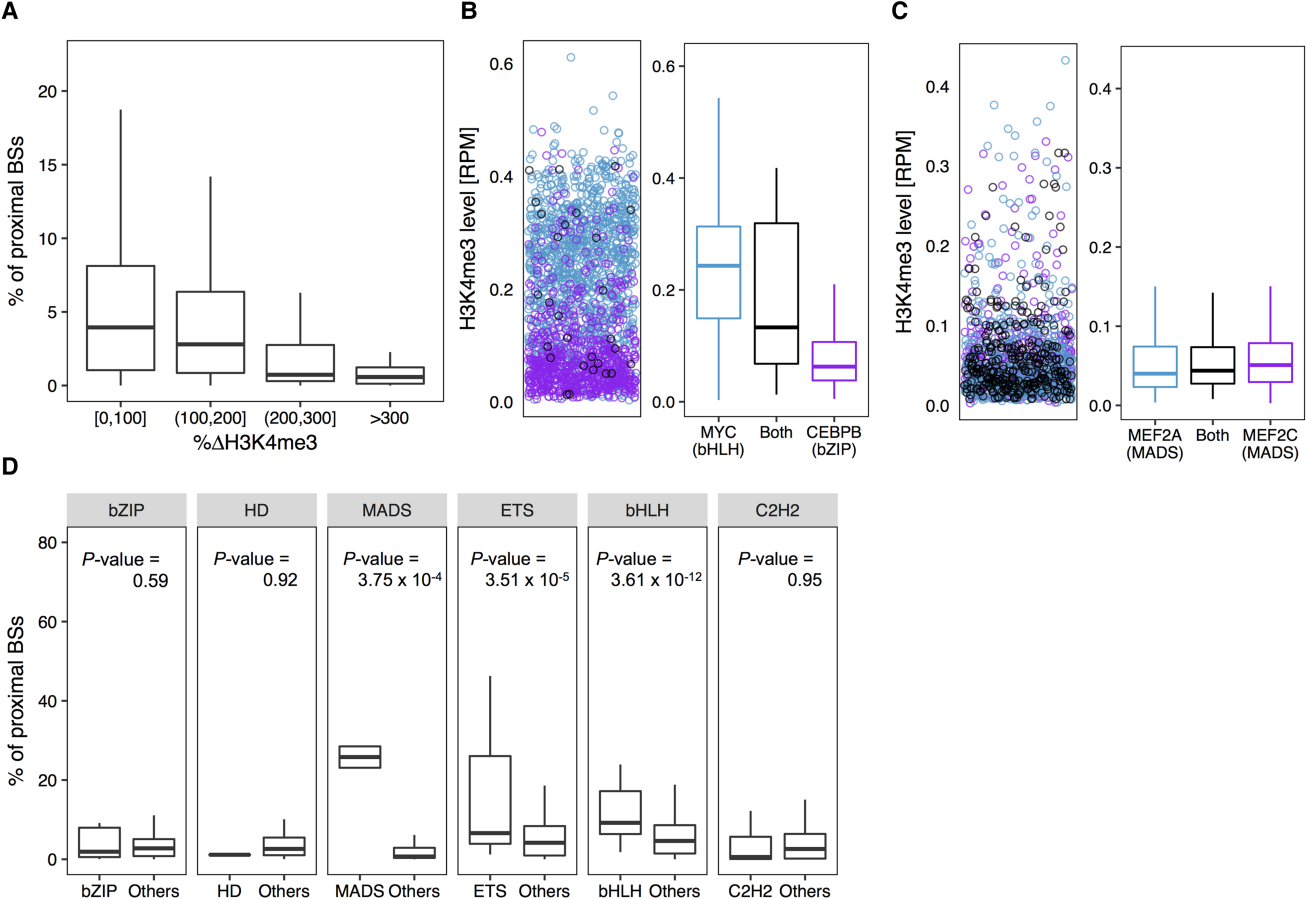
HM environment can constrain TF co-occupancy in the GM12878 cell line. TFs from the same protein family and TF families with a similarly favorable HM environment (or binding manner) tend to colocalize in the genome. (*A*) Box plots of percentages of BSs of a TF that are in close proximity (within 300 bp) to BSs of each of the other TFs versus average differences of H3K4me3 surrounding BSs between these two TFs. (*B*, *left*) H3K4me3 level surrounding BSs shared (black) by MYC (bHLH family) and CEBPB (bZIP family), MYC-only (blue), and CEBPB-only (purple). (*B*, *right*) Box plots representing the distribution of H3K4me3 levels surrounding BSs shared by MYC and CEBPB (black), MYC-only (blue), and CEBPB-only (purple). (*C*, *left*) H3K4me3 level surrounding BSs shared (black) by MEF2A and MEF2C (both from the MADS-domain family), MEF2A-only (blue), and MEF2C-only (purple). (*C*, *right*) Box plots representing the distribution of H3K4me3 levels surrounding BSs shared by MEF2A and MEF2C (black), MEF2A-only (blue), and MEF2C-only (purple). (*D*) Box plots displaying the distribution of percentages of proximal BSs among intra-family TF pairs and inter-family TF pairs for each protein family. One-sided Wilcoxon test *P*-values show that intra-family TF pairs have significantly higher percentages of proximal BSs compared to inter-family TF pairs.

To investigate this relationship, we calculated the co-occupancy of all possible TF pairs by measuring the percentage of proximal BSs between these pairs (Methods) and examined the distribution of H3K4me3 patterns surrounding their BSs. Presumably, intra-family TF pairs or pairs from protein families that use similar binding mechanisms (DNA sequence+shape or HM-specific manner) will prefer similar HM patterns surrounding their BSs. For these TF pairs, we would expect a tendency to colocalize and to exhibit more distinct TF co-occupancies compared to other characteristics. We found that TF pairs with similar H3K4me3 patterns around their BSs had a larger percentage of proximal BSs, and TF pairs with substantial H3K4me3 pattern differences tended to avoid binding closely to each other in the GM12878 cell line (Fig. 5A).

Here, we provide three examples of TF pairs to support this hypothesis. The first pair was MYC and CEBPB from the bHLH and bZIP families, respectively. In the GM12878 cell line, MYC showed HM-specific binding, whereas CEBPB preferred distinct DNA sequence and shape features (Fig. 4B). As a result, only ∼1% of the MYC BSs were proximal to CEBPB BSs, and CEBPB only shared ∼2% of its BSs with MYC. Furthermore, MYC-only BSs had higher H3K4me3 levels compared to CEBPB-only BSs (Fig. 5B). These observations were consistent with results in the K562 and H1-hESC cell lines (Supplemental Figs. S20, S21).

The second pair was MEF2A and MEF2C, both from the MADS-domain family. Both of these TFs bound DNA in an HM-specific manner. Even when we excluded overlaps of their BSs, ∼23% of MEF2A BSs were still proximal to MEF2C BSs, and ∼29% of MEF2C BSs were shared with MEF2A. Distributions of H3K4me3 levels surrounding MEF2A-only and MEF2C-only BSs were quite similar to levels surrounding MEF2A- and MEF2C-shared BSs (Fig. 5C).

The third example was of TF pairs from different TF families, wherein both TFs showed sequence+shape-specific DNA binding and similar H3K4me3 patterns surrounding their BSs. These pairs were NRF1 and EGR1 in the GM12878 cell line, NFYB and USF1 in the K562 cell line, and USF1 and JUND in the H1-hESC cell line (Supplemental Fig. S22). This observation indicates that TFs with similar preferences for HM patterns and similar binding mechanisms tend to co-occupy. Interestingly, the third TF pair had very different sequence preferences (Supplemental Fig. S4).

In agreement with these examples of TF pairs, we observed that intra-family TF pairs tended to have a significantly higher number of proximal BSs compared to inter-family TF pairs for protein families having consistent and different HM patterns around their in vivo BSs, such as the bHLH, MADS-domain, GATA, STAT, and ETS families (Fig. 5D; Supplemental Figs. S20, S21). In conclusion, our results suggest a close dependency between the HM pattern preferences of TFs and the tendency of TFs to occupy proximal BSs in vivo.

### Larger differences in HM patterns result in a substantial decrease in nucleosome occupancy

HMs can change DNA accessibility and nucleosome stability, either directly by adding methyl or acetyl groups to histone tails (Lu et al. 2008; Zentner and Henikoff 2013) or indirectly by recruiting specific proteins (e.g., with chromodomains at histone tails with methylation and bromodomains at histone tails with acetylation) (Bell et al. 2010; Canzio et al. 2011). Beyond these processes, HMs can affect nucleosome positioning (Anderson et al. 2001). Therefore, we hypothesized that HM patterns are closely related to in vivo TF binding specificities for certain protein families through their effects on nucleosome positioning.

**Figure 6.**
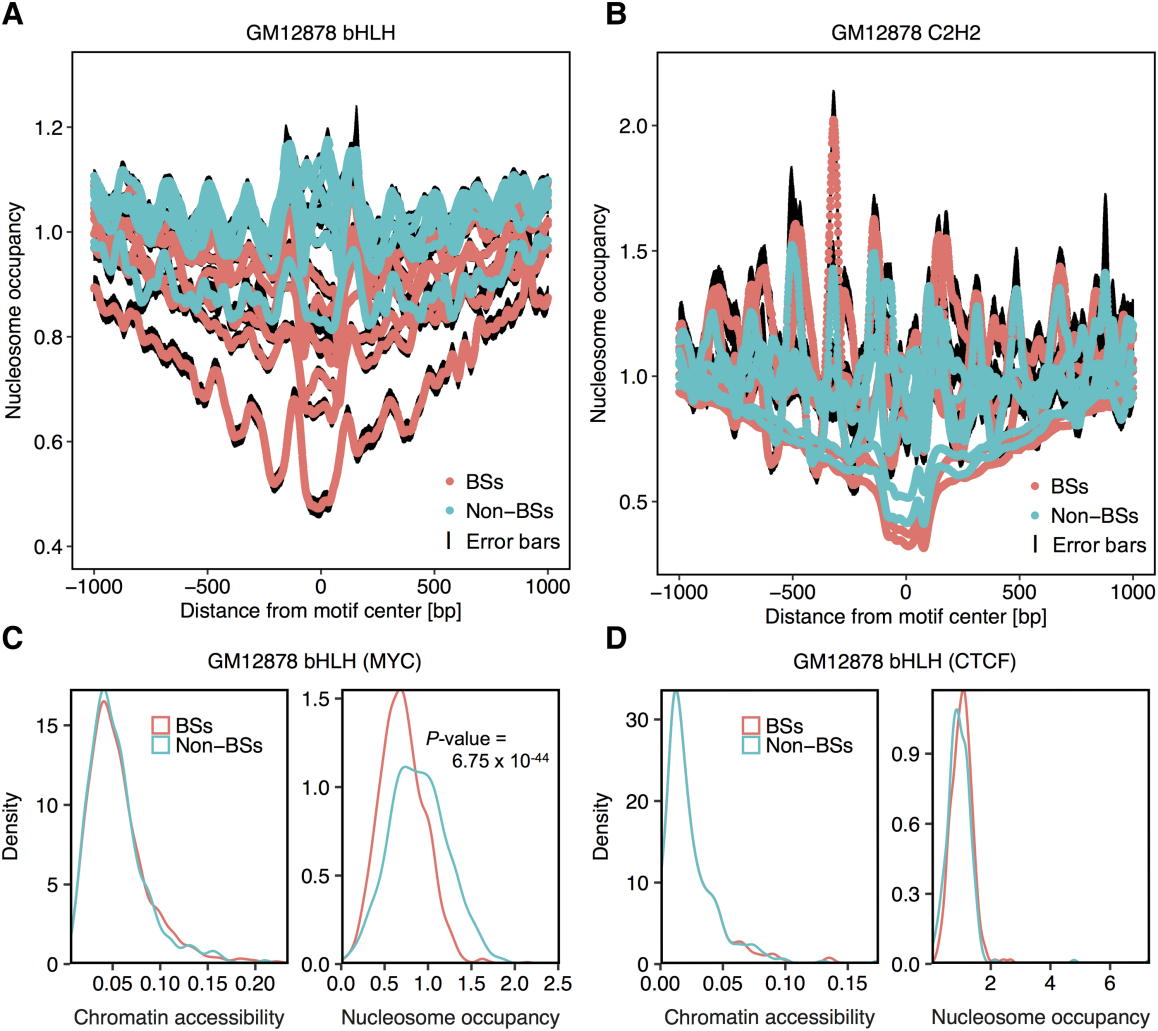
Nucleosome occupancy decreases around BSs compared to non-BSs among TF families that bind in an HM-specific manner. Average nucleosome occupancy in each position 1 kb upstream of and downstream from BSs and non-BSs for the bHLH (*A*) and C2H2 (*B*) families in the GM12878 cell line. Black edges encompassing the average line represent standard error bars at each nucleotide position. (*C*,*D*) Density plots showing distributions of chromatin accessibility and nucleosome occupancy around BSs and non-BSs for MYC in the bHLH family (*C*) and CTCF in the C2H2 family (*D*). Two-sided Wilcoxon tests were conducted to test if these distributions had shifts. Only distributions of nucleosome occupancy for MYC BSs exhibited significant shifts, as indicated by the *P-*value.

To test this hypothesis, we investigated the nucleosome positioning profiles surrounding BSs and non-BSs of TFs that bind in a DNA sequence+shape-specific and HM-specific manner. To derive nucleosome positioning profiles, we collected genome-wide MNase-seq data for the GM12878 and K562 cell lines from the ENCODE Project (Supplemental Table S1). We then derived the nucleosome occupancy at each base pair in 1-kb regions upstream of and downstream from known target sites. TFs from families with consistent HM patterns across cell lines and substantially different HM patterns between BSs and non-BSs (e.g., bHLH, ETS, GATA, and MADS-domain) exhibited substantial decreases in average nucleosome occupancy around their BSs (Fig. 6A; Supplemental Figs. S23, S24). These results indicated a competition between histones and TFs for target sites. In contrast, the extent of the average decrease in nucleosome occupancy in regions surrounding BSs was more diverse for TFs from the C2H2 family (Fig. 6B). For example, BSs of MYC exhibited lesser nucleosome occupancy than non-BSs (Fig. 6C), whereas the nucleosome occupancy distributions of BSs and non-BSs of CTCF were similar (Fig. 6D). BSs of other TFs in the bHLH family displayed more substantially decreased nucleosome occupancy than BSs of other TFs in the C2H2 family (Supplemental Figs. S25, S26). In our experimental setup, BSs and non-BSs of each TF had similar distributions of chromatin accessibility.

Considering that nucleosome occupancy might be a more direct factor affecting TF binding than HM patterns, we implemented additional L2-regularized MLR models (Methods) using a combination of DNA sequence and shape, and average nucleosome occupancy to classify BSs and non-BSs. With consistently increased performance compared to sequence+shape models across cell lines, nucleosome occupancy (nuc) can contribute to the distinction of in vivo BSs and non-BSs (Supplemental Figs. S27, S28). Because HM patterns contain information for both nucleosome positioning and HM levels, sequence+shape+HM models generally outperformed sequence+shape+nuc models because nucleosome occupancy was represented by only one feature compared to 10 HM features (Supplemental Fig. S29). Interestingly, sequence+shape+nuc models were more sensitive to flanking length than were sequence+shape+HM models, indicating that nucleosome occupancy is more accurate in describing local chromatin structure as it is relevant to TF binding. These observations indicate that HM-selective TFs require increased nucleosome positioning flexibility compared to TFs that bind in a DNA sequence+shape-dependent manner.

## Discussion

DNA sequence and shape preferences at flanking regions of core motifs play important roles in achieving binding specificity for TFs from certain protein families, both in vitro (Gordân et al. 2013; Afek et al. 2014) and in vivo (Dror et al. 2015). However, these DNA properties alone are insufficient to explain the different in vivo TF binding preferences observed across distinct cell types (The ENCODE Project Consortium 2012; Ernst and Kellis 2013; Tsankov et al. 2015). Considering cell-type–specific properties, previous studies described a general relationship between TF binding and HM patterns (Guccione et al. 2006; Arnold et al. 2013; Grubert et al. 2015; Ziller et al. 2015). These studies reported increased performances in the sequence-based modeling of in vivo TF binding events when such models incorporated HMs (Benveniste et al. 2014; Liu et al. 2015). In this study, we described qualitatively and quantitatively the relationship between TF binding and HM patterns in a protein family–specific manner across different cell lines. We revealed that TFs from certain TF families displayed conserved HM pattern preferences between BSs and non-BSs.

To investigate preferences in HM patterns surrounding BSs compared to non-BSs, we analyzed comprehensive ENCODE data (The ENCODE Project Consortium 2012) and examined in vivo ChIP-seq data for 33, 37, and 18 TFs in the GM12878, K562, and H1-hESC cell lines, respectively. The studied TFs covered eight protein families, including the C2H2, MADS-domain, bHLH, bZIP, HD, STAT, GATA, and ETS families. Among the feature categories in flanking regions of core motifs that influence in vivo TF binding, two important determinants are chromatin accessibility (Song et al. 2011) and DNA sequence context (Levo and Segal 2014). Closed chromatin is inaccessible to most TFs, whereas open chromatin provides accessible regions for TF binding that are generally transcriptionally active (Grewal and Moazed 2003; Huisinga et al. 2006). Considering these mechanisms, we restricted our data set of non-BSs to exactly matched core motifs with a similar distribution of chromatin accessibility as in environments of BSs. Then, we examined the differences in HM patterns between regions surrounding BSs versus non-BSs.

In regions surrounding BSs, preferences for HM patterns were protein family specific. TFs from the bHLH, GATA, and MADS-domain families displayed, across cell lines, decreased levels of repressive histone marks (such as H3K27me3 modification) and increased levels of active histone marks (such as H3K4me1, H3K27ac, H3K4me3, and H3K79me3 marks) (Shlyueva et al. 2014). The C2H2, bZIP, and HD families, however, showed more divergent HM patterns surrounding their BSs. As one of the largest TF families in eukaryotes, the C2H2 family exhibited varying preferences, likely due to their much less conserved three-dimensional protein folds compared to other protein families (Laity et al. 2001). Besides the 10 HM patterns considered, our work can be further extended by adding other histone marks on linker histones, which closely relate to chromatin structure and TF binding (Fyodorov et al. 2017), as well as additional HM marks in core histones.

Recent studies reported a quantitative relationship between in vivo TF binding specificities and HM patterns surrounding BSs near DNA regulatory elements (Benveniste et al. 2014; Liu et al. 2015). Given our qualitative observation that HM pattern preferences surrounding BSs are protein family specific, an obvious question is whether HM patterns at TF BSs can add another layer to modeling genome-wide in vivo TF binding quantitatively in a protein family–specific manner. Using DNA sequence and shape profiles as feature categories in our baseline models, HM-augmented models for predicting TF binding resulted in larger performance increases for TFs from protein families that had substantial HM pattern differences between BSs and non-BSs, such as members of the bHLH, MADS-domain, and GATA families. This result indicates that HM patterns may contribute to the binding of TFs from the same protein family to their putative BSs. Furthermore, other mechanisms, such as cofactors, cooperativity, and higher-order chromatin structure, could further increase the quantitative modeling of TF binding in vivo.

We previously suggested that specific TF families use different contributions of DNA sequence and shape at flanking regions to achieve binding specificities in vivo and in vitro (Dror et al. 2015). Furthermore, it is well established that eukaryotic transcriptional regulation requires many layers of binding specificity determinants (Lelli et al. 2012). Thus, here we disentangled the contributions of DNA sequence and shape profiles and HM patterns in distinguishing BSs from non-BSs based on HM-augmented binding specificity models. We found that contributions from these two sources were complementary to each other. We further identified three binding mechanisms: sequence+shape specific, HM specific, and a group with other features preferred. For most TFs from the bHLH, ETS, GATA, and MADS-domain families, our data suggest an HM-dependent binding mode. In contrast, TFs from the bZIP and C2H2 families seem to utilize a combination of sequence+shape-specific and HM-specific modes. Moreover, we conducted leave-one-feature-out feature selection experiments to deconvolve the contribution of each individual feature rather than of a set of features. Certain feature importance results validated our previous understanding of TF binding and might provide further insights into the role of other features in a systematic way. These observations were consistent for all three considered cell lines.

TFs tend to cobind DNA in close vicinity to each other in order to regulate transcriptional processes cooperatively. Our analysis revealed a dependency between the HM pattern preferences of TF pairs and their tendency to cobind the genome, even if they have different DNA sequence preferences. This interdependency indicates that HM patterns in regions where BSs are located constrain TF co-occupancy. TFs from the same protein family, or TFs from different TF families that bind in an HM-specific manner, tend to cobind DNA BSs in close proximity. Hox proteins from the HD family, for instance, bind in close proximity to their cofactors from the same protein family to unleash their DNA binding specificities (Slattery et al. 2011; Abe et al. 2015). It is possible that HM patterns explain the observation that cooperativity can occur promiscuously between TFs from diverse structural families (Jolma et al. 2015).

Given our observation that HM patterns contribute to the quantitative modeling of TF binding specificities, an intriguing question is how TFs can sample the unique HM environment far beyond the core motif in vivo. Other studies suggested that HMs have a direct physical effect on chromatin structure (Shogren-Knaak et al. 2006). Lysine acetylation, for instance, removes the positive charge of this residue, which is believed to increase DNA negative supercoiling (Rothbart and Strahl 2014). In another example, DNA topology is intricately connected with nucleosome structure and stability (Gupta et al. 2009). These DNA topological changes might influence the binding of regulatory proteins to DNA (Kouzine et al. 2013). Moreover, HMs modulate the nucleosomal barriers of the transcriptional machinery by altering histone-DNA contacts so that transcription proceeds efficiently (Teves et al. 2014). Considering these hypotheses, we examined the nucleosome occupancy surrounding BSs and non-BSs for various TF families. Protein families with larger HM pattern differences tended to exhibit decreased nucleosome occupancy at BSs compared to non-BSs. This finding indicates that HM pattern changes influence nucleosome structure and stability, which in turn, evoke changes in TF binding events. With the observation that the readout of regulatory sequences might be affected by TF–nucleosome interactions (Levo et al. 2017), future studies will be required to examine a possible HM–TF interplay.

In summary, with stringent experimental setups, our analysis extends current knowledge about the close relationship between HM patterns and genome-wide in vivo TF binding specificities by revealing protein family–specific mechanisms. We found that HM pattern differences surrounding BSs and non-BSs are TF family dependent, and that the contribution of HM patterns to quantitative models of binding specificities is TF family specific across different cell lines.

## Methods

### In vivo data collection and motif alignment

ChIP-seq data for human TFs with position frequency matrices (PFMs) in the JASPAR database (Mathelier et al. 2016a) and DNase I hypersensitivity sites were downloaded from The ENCODE Project Consortium (2012). Based on these PFMs, we used FIMO (Grant et al. 2011) to search and align BSs with default settings. If a motif was found more than once in a sequence, then the motif with lowest FIMO *P*-value was used. Data for the TF was discarded if (1) the number of aligned BSs was less than 132, to avoid the risk that the sample size would be less than the number of features used in the downstream MLR models (which have a minimum of 80 features); or (2) the peak of the motif distribution did not coincide with the ChIP-seq peak summit. Final numbers of data sets for the GM12878, K562, and H1-hESC cell lines were 33, 37, and 18 TFs, respectively.

TF data sets in each cell line were assigned to TF families according to the JASPAR database (Mathelier et al. 2016a). TF families with fewer than two members were grouped under “Other.” BSs were derived from ChIP-seq peaks.

### Background definition

For each BS, Bowtie (Langmead et al. 2009) was used to scan exact-matched sequences at chromatin-accessible regions determined by DNase-seq experiments. If more than one exact-matched sequence was found at a chromatin-accessible region, then only the first sequence in the Bowtie output was kept. Non-BSs were selected at distinct genomic locations with BSs. After these steps, for each BS, we selected one non-BS that had the closest average chromatin accessibility surrounding 1-kb regions upstream of and downstream from this BS. The resulting non-BSs had similar chromatin accessibility distributions and similar sample sizes as the selected BSs. Imbalanced data were generated by resampling five times non-BSs with a bootstrapping strategy.

### HM patterns of motif environments

As the motif environment, we considered 1-kb genomic regions upstream of and downstream from each motif. Based on the ChIP-seq BAM files for each HM, BEDTools suite *coverage* (Quinlan and Hall 2010) was performed to calculate the coverage of each nucleotide as the number of reads that included a given nucleotide. The HM level in each motif environment was averaged over the coverage at the core motif and 1-kb genomic regions surrounding the core motif (i.e., 2 kb + motif length). Then, the HM level was normalized by computing the value of reads per million (RPM). Average levels were taken from experimental replicates.

### DNA shape features in flanking regions

Starting from motifs in BSs and non-BSs, sequences at 10-bp regions upstream of and downstream from the motifs were extracted. To utilize DNA shape profiles at each nucleotide of these sequences, these sequences with 2-bp flanking regions were generated as input for DNAshapeR (Chiu et al. 2016), our R software package for high-throughput DNA shape feature prediction. Four DNA structural features (i.e., MGW, ProT, Roll, and HelT) were calculated, among which MGW and ProT were predicted for each nucleotide position, and Roll and HelT were predicted for each base pair step of these sequences.

### Statistical comparison of HM patterns at bound BSs and non-BSs

We compared HM patterns between BS and non-BS environments for each HM using the one-sided Wilcoxon signed rank test implemented by *wilcox.test* in R. The option *greater* in the test meant the null hypothesis (BSs > non-BSs) and vice versa. Bonferroni correction was applied to correct for multiple testing. The *Q*-values corrected from tests with the *greater* and the *less* options were used to calculate Δ[−log(*q*-value)], which indicates the results in HM pattern comparisons between BSs and non-BSs. A positive Δ[−log(*q*-value)] was assigned to a HM when this HM surrounding BSs had significantly higher levels than surrounding non-BSs, and vice versa.

### MLR scoring scheme

Three different L2-regularized MLR models were trained to distinguish BSs from non-BSs by using the following feature combinations: (1) DNA sequence and four DNA shape features (MGW, Roll, ProT, and HelT) at flanking regions 5′ and 3′ of the core motif; (2) DNA sequence and four DNA shape features at flanking regions, and 10 HM patterns at the core motif (usually 6–20 bp) and 1-kb regions upstream and downstream; and (3) DNA sequence and four DNA shape features at flanking regions 5′ and 3′ of the core motif, and nucleosome occupancy at the core motif and 1-kb regions upstream and downstream. For each BS, DNA sequence was represented in a feature vector (where A was encoded as 1000, T as 0100, G as 0010, and C as 0001). Training sets for MLR classification were stacks of BSs (labeled as “1”) and non-BSs (labeled as “0”). The penalty parameter λ was learned from the data by using an embedded 10-fold cross-validation on the training set. AUPRC, computed by using the ROCR package in R (Sing et al. 2005; R Core Team 2015), was used to evaluate the accuracy of the respective models in predicting BSs and non-BSs.

### Leave-one-feature-out L2-regularized MLR models

To determine the importance of each feature in the classification models combining DNA sequence, shape, and HM features, we implemented MLR models in which we left out one feature at a time (i.e., DNA sequence, MGW, ProT, Roll, HelT, H3K4me2, H3K27ac, H3K4me1, H3K4me3, H3K79me2, H3K9ac, H3K9me3, H4K20me1, H3K27me3, and H3K36me3). We recorded the percentage decrease of AUPRC for each altered model compared to models that considered all features.

### Calculating co-occupancy of a TF pair

The percentage of proximal BSs of all possible TF pairs was calculated in each of the three cell lines. Proximal BSs for a TF pair were defined similarly to Dror et al. (2015). All ChIP-seq peaks containing BSs of a given TF were collected and extended 300 bp at each flank. We calculated the percentage of proximal BSs for each TF pair by examining the number of BSs of the TF pair that were located inside these extended peaks. Because TFs from the same family usually have similar preferences for genomic sequences, we discarded overlapping BSs. We measured the percentage of ΔH3K4me3 for each TF pair by the difference ratio of the average H3K4me3 patterns over the environment of all BSs. Last, for each TF pair, we compared the percentage of proximal BSs to the H3K4me3 pattern difference ratio around the BSs.

### Nucleosome occupancy

Genome-wide MNase-seq data for the GM12878 and K562 cell lines were downloaded from The ENCODE Project Consortium (2012) in bigWig format. BS and non-BS coordinates were derived from our MLR classification model. Nucleosome occupancy at base pair resolution was calculated by bwtool, developed by Pohl and Beato (2014). For each TF, we calculated the average nucleosome occupancy for regions 1 kb upstream of and downstream from all BSs and non-BSs (i.e., 2 kb + motif length).

### Software availability

Source code implementing data preprocessing and L2-regularized MLR models, as well as BSs and non-BSs in the GM12878 cell line, are available in the GitHub repository at https://github.com/xinbeibei/HM_and_TFbinding and in Supplemental Material.

## Supplementary Material

Supplemental Material
