# Supplementary material for: Relationship between histone modifications and transcription factor binding is protein family specific
Source: Genome Res. 2018 Mar;28(3):321–33. doi: 10.1101/gr.220079.116 (PMC5848611; doi:10.1101/gr.220079.116)
Supplement: Supplemental Material [file supp_28_3_321__index.html]

Relationship between histone modifications and transcription factor binding is protein family specific — Supplemental Material 

# Relationship between histone modifications and transcription factor binding is protein family specific

## Supplemental Material

- Supplemental\_Methods.pdf
- Supplemental\_Figures\_S1-29.pdf
- Supplemental\_Tables\_S1-4.xlsx
- Supplemental\_Material.zip
