# Supplementary material for: Relationship between histone modifications and transcription factor binding is protein family specific
Source: Genome Res. 2018 Mar;28(3):321–33. doi: 10.1101/gr.220079.116 (PMC5848611; doi:10.1101/gr.220079.116)
Supplement: Supplemental Material [file supp_gr.220079.116_Supplemental_Methods.pdf]

SUPPLEMENTAL METHODS

Relationship between histone modifications and transcription factor binding is protein family specific

Beibei Xin and Remo Rohs*

Computational Biology and Bioinformatics Program, Departments of Biological Sciences, Chemistry, Physics & Astronomy, and Computer Science, University of Southern California, Los Angeles, CA 90089, USA

*Correspondence: rohs@usc.edu

Procedures for obtaining binding sites, modeling, and prediction accuracy assessment

In this study, our focus was to discriminate in vivo binding sites (BSs) and non-BSs with exactly-matched core motifs and similar chromatin accessibility at flanking regions. For each transcription factor (TF) in each cell line, BSs and non-BSs used for statistical analysis and machine learning were obtained by the following steps:

1. Download ChIP-seq peaks in *.narrowPeak* format from the ENCODE Project (Supplemental Table S1; http://genome.ucsc.edu/ENCODE/downloads.html) as sequences containing potential BSs. Download DNase-seq data in *.narrowPeak* format from the ENCODE Project (Supplemental Table S1) for chromatin-accessible regions as sequences containing potential non-BSs.

2. Download position frequency matrices (PFMs) from the JASPAR database (Mathelier et al. 2015). Convert each PFM into a position weight matrix (PWM) and apply this PWM as the binding profile in FIMO (Grant et al. 2011). Scan motifs in the ChIP-seq peaks (resulting in set 1) with default settings (*P*-value = 0.0001). Calculate the binding energy in Supplemental Fig. S5 for each TF as the average of PWM scores for sequences in BSs. Given a PWM $P_{4 \cdot L}$, log likelihood

is calculated to convert P into a matrix M. Then, the estimated binding energy of each sequence S is calculated by the following formula:

$$M_{k,j} = log_2(P_{k,j}/0.25);\ Score(S) = \sum_{j=1}^{L}\sum_{k=A}^{T} M_{k,j} I_{(S_j=k)},\ k=A,C,G,T.$$

3. For non-BSs, remove accessible regions that overlap with set 1 and use Bowtie (Langmead et al. 2009) to find an exactly matched sequence for each sequence in set 1 from the remaining accessible regions, resulting in set 2. Sets 1 and 2 are not overlapping and have exactly matched core motifs. However, because sets 1 and 2 may have imbalanced sample sizes, we downloaded DNase-seq data in *.bigWig* format (Supplementary Table S1), used *bwtool aggregate* to calculate average chromatin accessibility in 1 kb surrounding sequences in both sets, sampled sequences from the set with more sequences, and ensured that the sets had similar sample sizes and chromatin accessibility distributions (Supplemental Fig. S1). After this step, BSs and non-BSs were generated.

4. Check sample size and consistency of peak centers and motif centers to decide whether to discard this TF or not. Keep a TF if: (i) the number of BSs is larger than 132, to avoid the risk that the sample size would be less than the number of features used in downstream MLR models (which have a minimum of 80 features); and (ii) the peak of the motif distribution coincides with the ChIP-seq peak summit. For each motif in the BSs, calculate the distance from the motif center to peak summit of the ChIP-seq peak region where the motif is located. Draw a distribution/histogram of these distances over all motifs in the BSs of a TF. If the distribution has a peak at distance 0, then the TF was kept in the dataset. A similar strategy was previously used by (Dror et al. 2015).

5. For each motif in BSs and non-BSs, encode sequence information at flanking regions into binary numbers and calculate the four DNA shape features minor groove width (MGW), Propeller Twist (ProT), Roll, and Helix Twist (HelT) surrounding core motifs using DNAshapeR (Chiu et al. 2015). Normalize DNA shape features independently by using the equation:

$$norm_{value} = (value - min_{global})/std_{global}$$

where $norm_{value}$ is the normalized value to compute, *value* is the DNA shape feature value, and min_{global} (respectively std_{global}) corresponds to the minimum (respective standard deviation) possible value across all pentamers probable based on this high-throughput method. Concatenate histone modification (HM) patterns around each motif with DNA sequence and shape features for the motif. Obtain the feature vector as the input for the machine-learning model.

6. Apply L2-regularized multiple linear regression (MLR) models to classify BSs (label 1) and non-BSs (label 0) using an embedded 10-fold cross-validation on the training set. Motifs with predicted response variable (label) larger than 0.5 are assigned label 1. Compute the area under the precision-recall curve (AUPRC) with *ROCR* package in R (Sing et al. 2005; R Core Team. 2015).
