# Supplementary material for: Relationship between histone modifications and transcription factor binding is protein family specific
Source: Genome Res. 2018 Mar;28(3):321–33. doi: 10.1101/gr.220079.116 (PMC5848611; doi:10.1101/gr.220079.116)
Supplement: Supplemental Material [file supp_gr.220079.116_Supplemental_Figures_S1-29.pdf]

SUPPLEMENTAL FIGURES

Relationship between histone modifications and transcription factor binding is protein family specific

Beibei Xin and Remo Rohs*

Computational Biology and Bioinformatics Program, Departments of Biological Sciences, Chemistry, Physics & Astronomy, and Computer Science, University of Southern California, Los Angeles, CA 90089, USA

*Correspondence: rohs@usc.edu

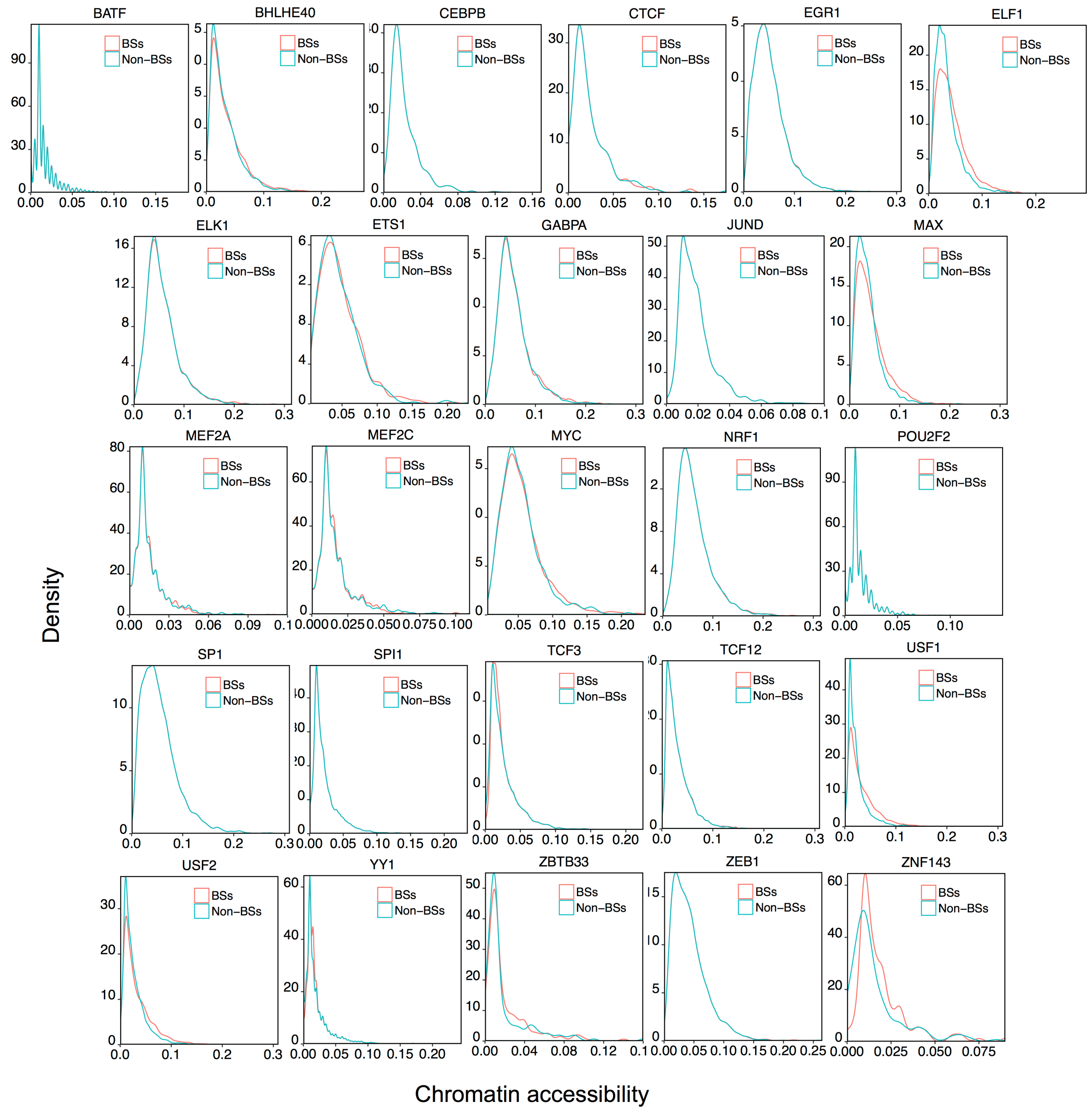

Supplemental Fig. S1. Distributions of average chromatin accessibility at regions 1 kb upstream and downstream of BSs and non-BSs for TFs in the GM12878 cell line. Here, we display TFs from all TF families except for unassigned datasets ("other" designation).

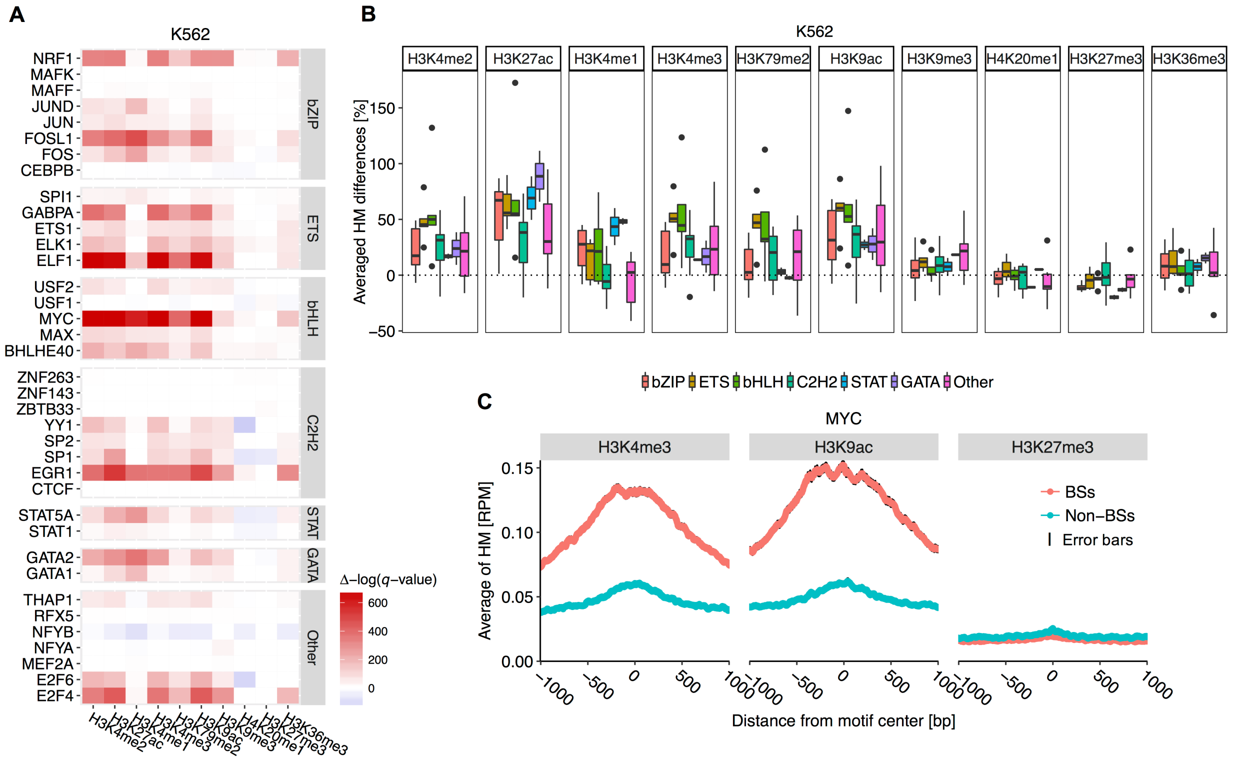

Supplemental Fig. S2. TF families show conserved differences in HM patterns between BSs and non-BSs. (A) Heat map displaying results of statistical comparison between HM levels at positions 1 kb upstream and downstream of BSs and non-BSs in the K562 cell line. Positive Δ(–log(*q*-value)), in red, indicates BS environments with significantly higher HM levels compared to non-BS environments. Negative Δ(–log(*q*-value)), in blue, indicates BS environments with lower HM levels. The C2H2, ETS, and bHLH TF families show conserved HM pattern differences. (B) Average HM differences across TF families in the K562 cell line. Centerlines of box plots represent medians, edges represent first and third quartiles, and whiskers indicate minimum/ maximum values within 1.5 times the interquartile from the box. (C) Average H3K4me3, H3K9ac, and H3K27me3 levels at each position 1 kb upstream and downstream of BSs and non-BSs for MYC (bHLH TF family). Black edges encompassing the average line represent standard error bars at each nucleotide position.

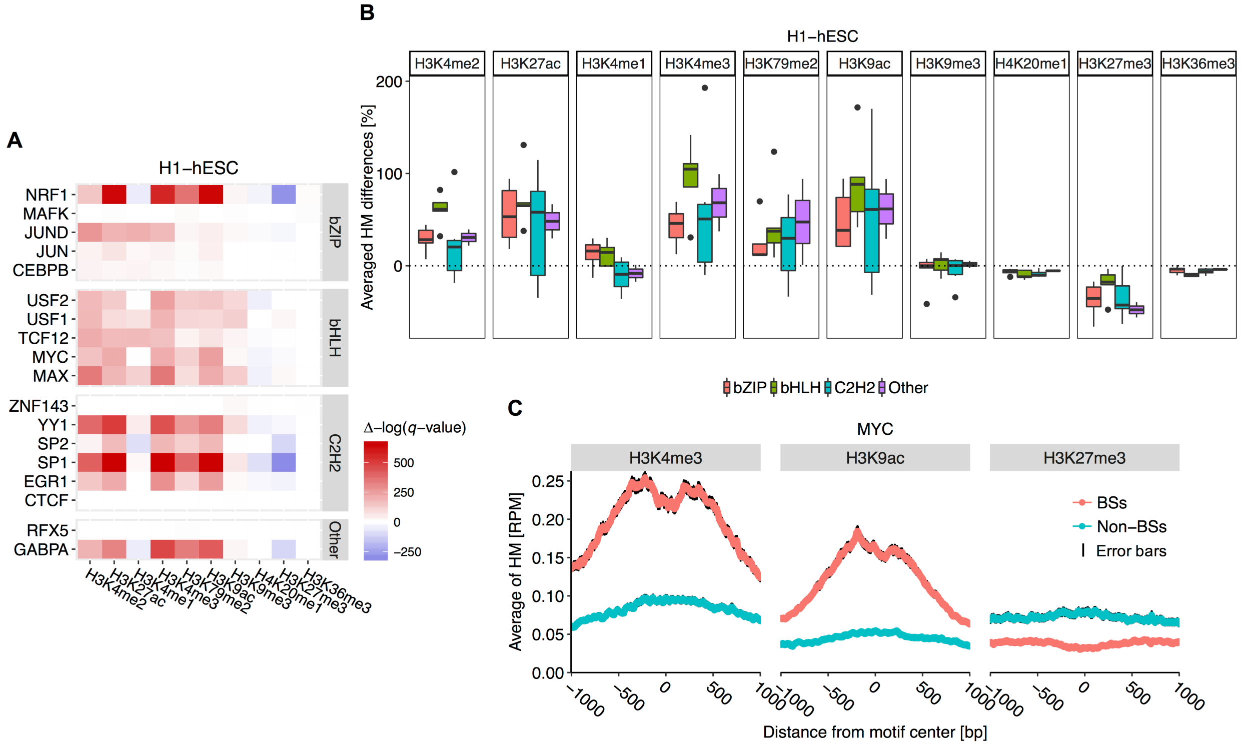

Supplemental Fig. S3. TF families show conserved differences in HM patterns between BSs and non-BSs. (A) Heat map displaying results of statistical comparison between HM levels at positions 1 kb upstream and downstream of BSs and non-BSs in the H1-hESC cell line. Positive Δ(–log(*q*-value)), in red, indicates BS environments with significantly higher HM levels compared to non-BS environments. Negative Δ(–log(*q*-value)), in blue, indicates BS environments with lower HM levels. C2H2 and bHLH TF families show conserved HM pattern differences. (B) Average HM differences across TF families in the H1-hESC cell line. Centerlines of box plots represent medians, edges represent first and third quartiles, and whiskers indicate minimum/ maximum values within 1.5 times the interquartile from the box. (C) Average H3K4me3, H3K9ac, and H3K27me3 levels at each position 1 kb upstream and downstream of BSs and non-BSs for MYC (bHLH family). Black edges encompassing the average line represent standard error bars at each nucleotide position.

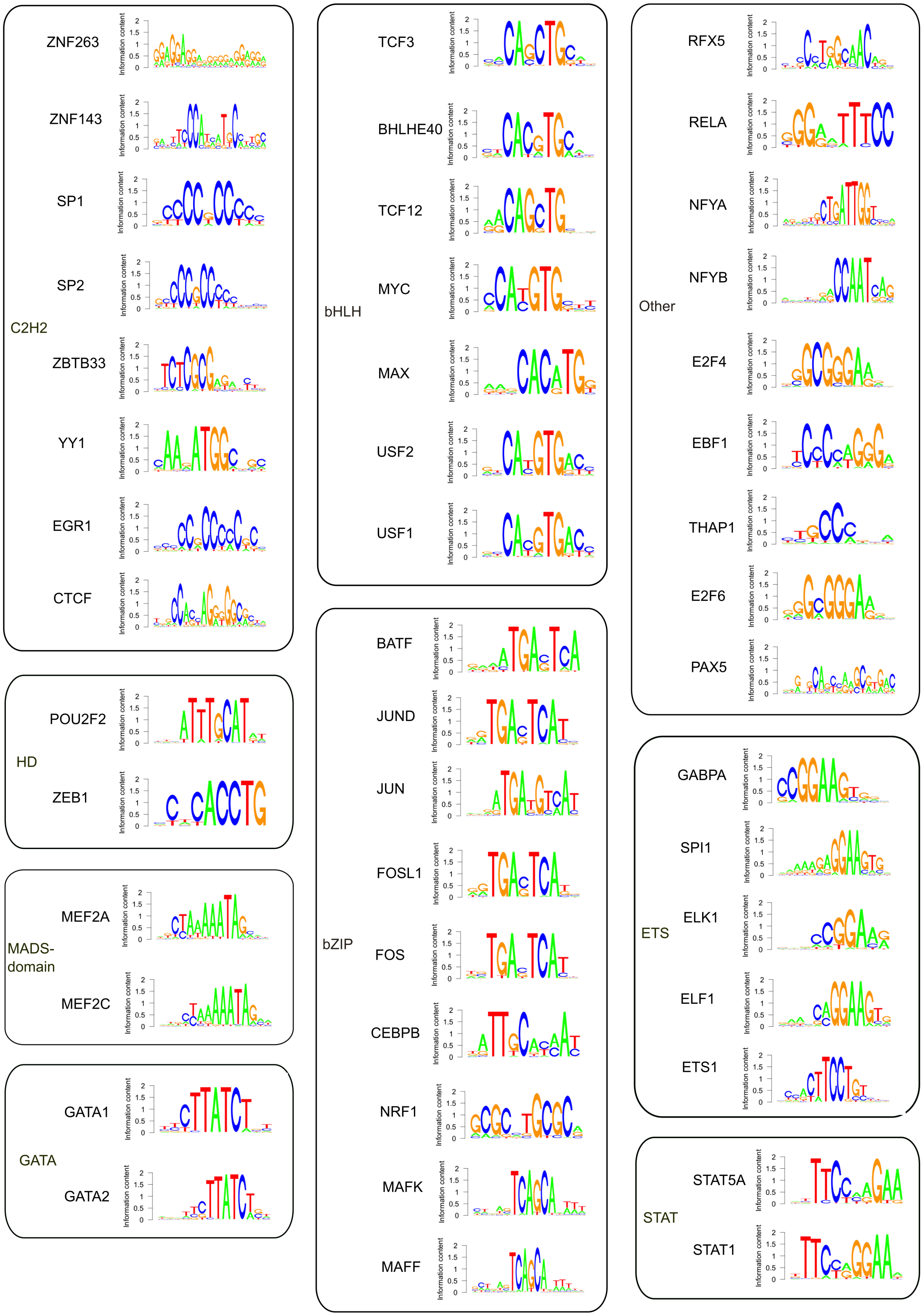

Supplemental Fig. S4. Visualize PWMs downloaded from the JASPAR database (Mathelier et al. 2016) for all TFs studied in three cell lines.

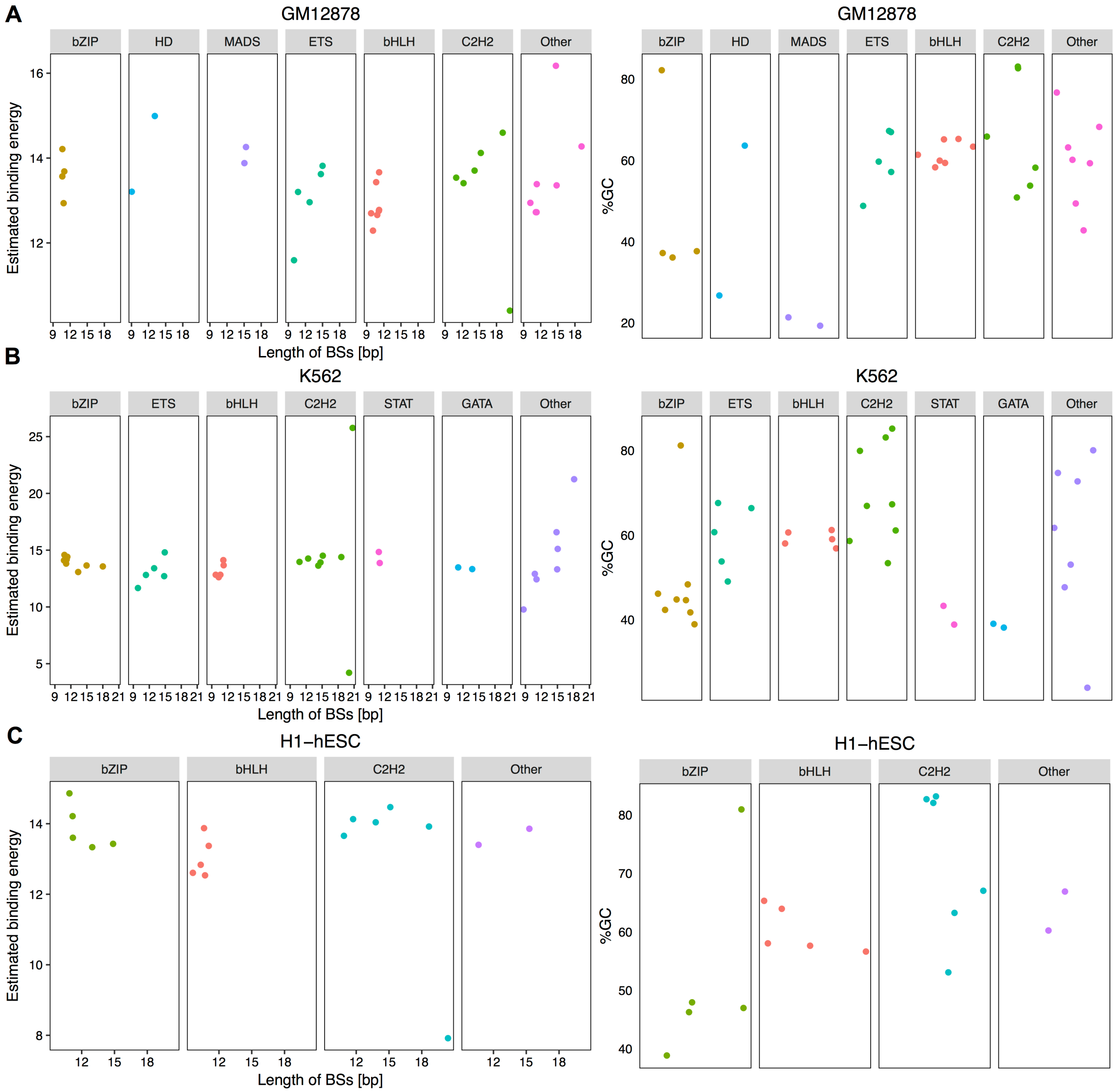

Supplemental Fig. S5. (A) Jitter plots showing estimated binding energy for each TF in all three cell lines corresponding to motif length of the TF. Estimated binding energy was calculated as the average of PWM scores for BSs. (B) Jitter plots displaying different GC content for each TF in all three cell lines.

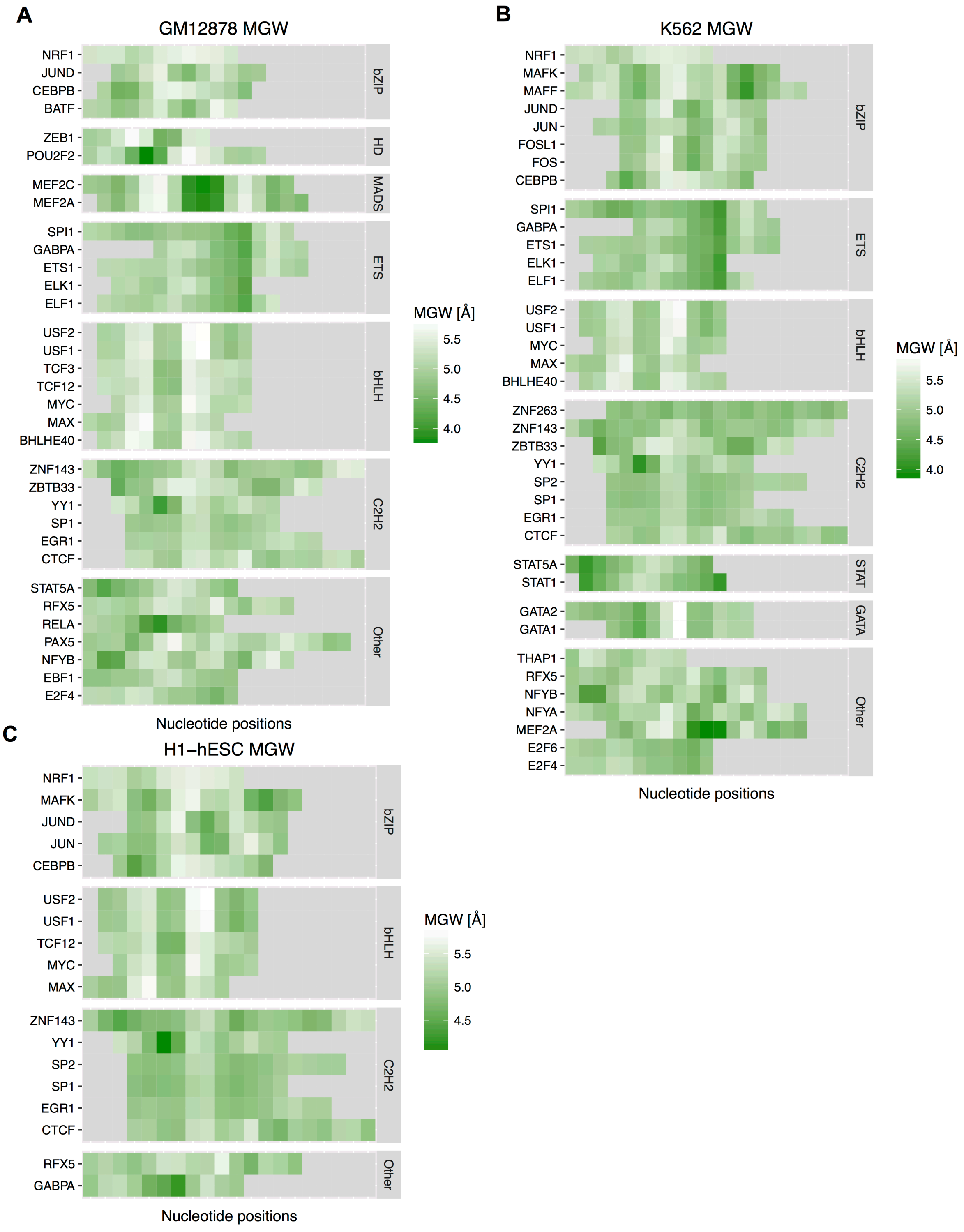

Supplemental Fig. S6. Heat maps displaying average MGW features around BSs of TFs in the (A) GM12878, (B) K562, and (C) H1-hESC cell lines.

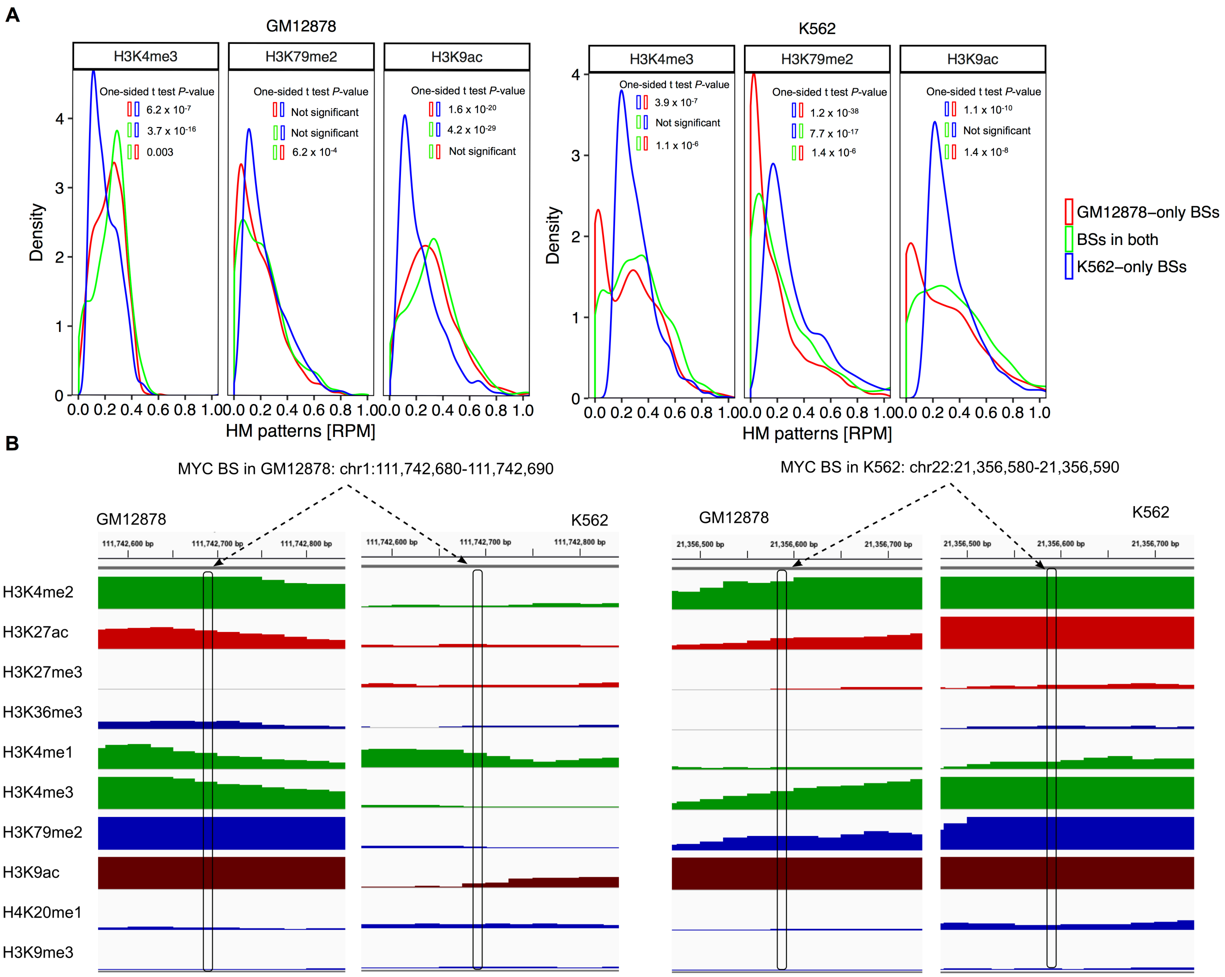

Supplemental Fig. S7. MYC BSs in both GM12878 and K562 cell lines were merged and partitioned into three groups: GM12878-only BSs, BSs in both, and K562-only BSs. (A) HM patterns in GM12878 and K562 cell lines of these groups of MYC BSs are visualized. For one-sided t-tests, the alternative hypothesis is that the group denoted by the box on the left is larger than the group denoted by the box on the right. (B) Comparison of 10 HM patterns around one MYC BS in the GM12878 cell line and one MYC BS in the K562 cell line.

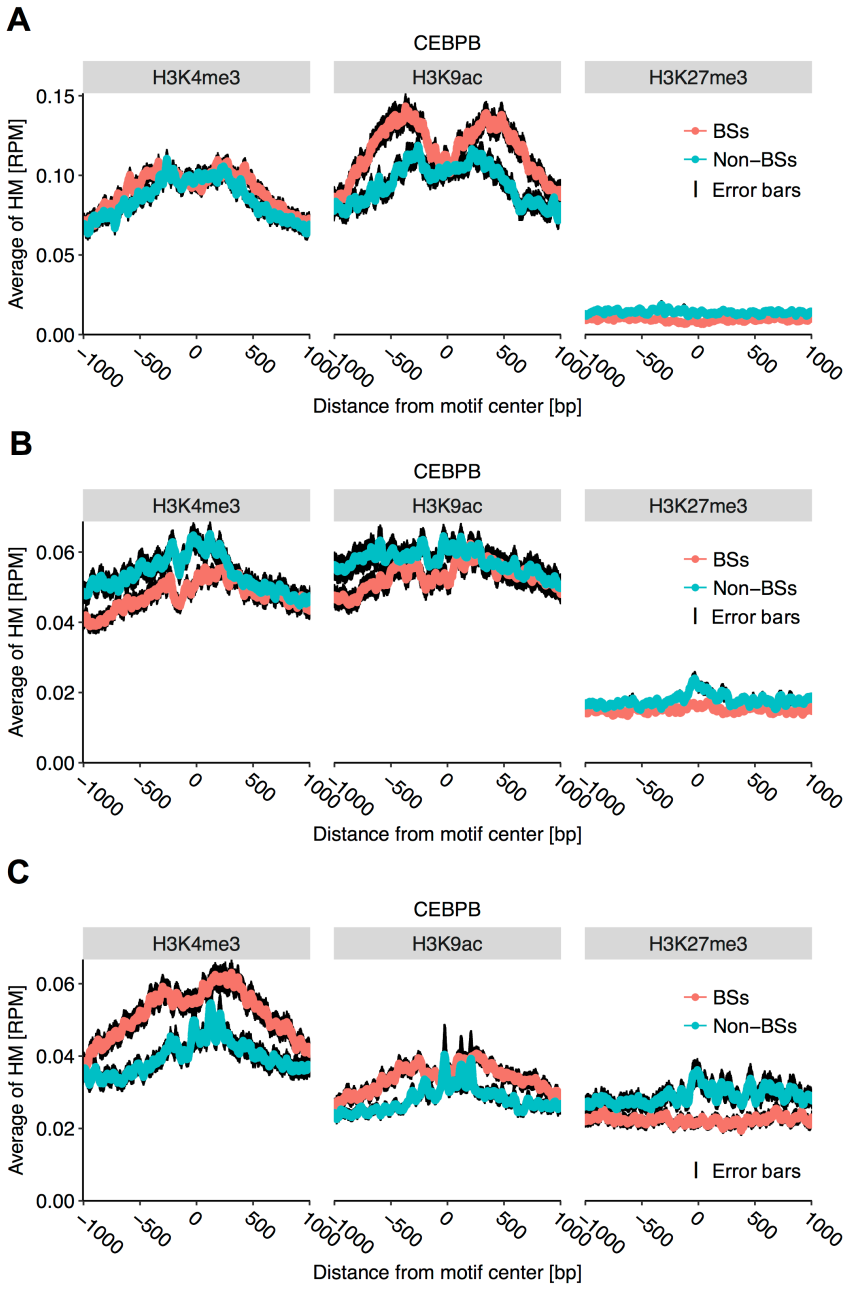

Supplemental Fig. S8. Average H3K4me3, H3K9ac, and H3K27me3 levels in each position 1 kb upstream and downstream of BSs and non-BSs for CEBPB (bZIP TF family) in the (A) GM12878, (B) K562, and (C) H1-hESC cell lines. Black edges around average lines are standard error bars at each position.

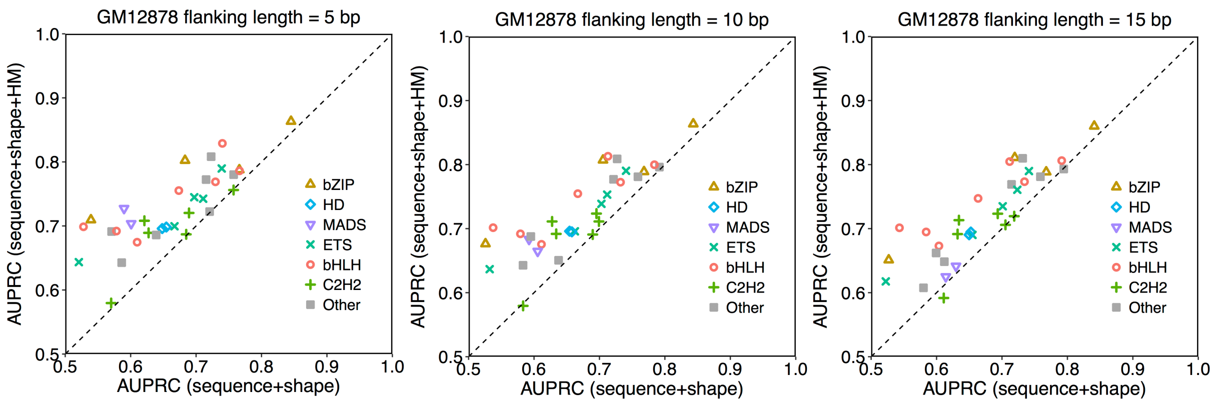

Supplemental Fig. S9. Different length scales of flanking regions in calculating DNA sequence and shape features and their effects on quantitative prediction of in vivo TF binding in the GM12878 cell line.

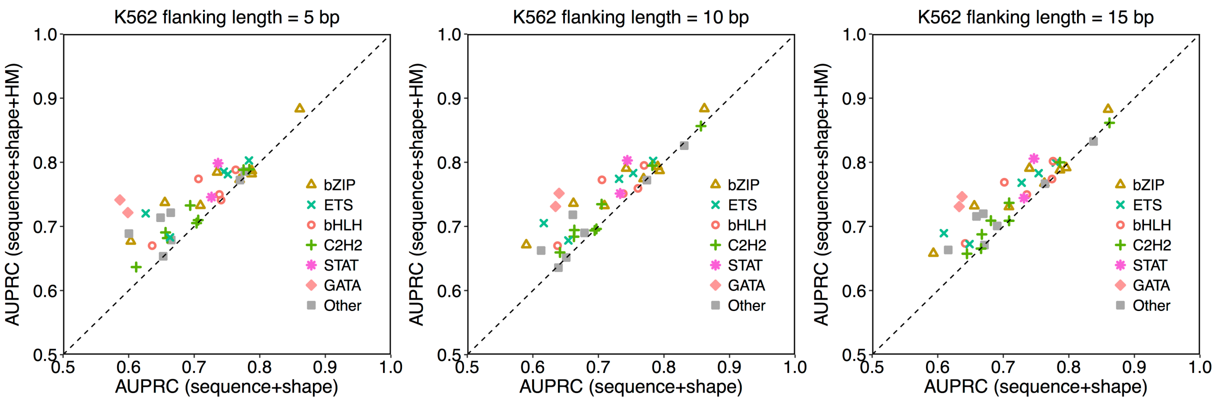

Supplemental Fig. S10. Different length scales of flanking regions in calculating DNA sequence and shape features and their effects on quantitative prediction of in vivo TF binding in the K562 cell line.

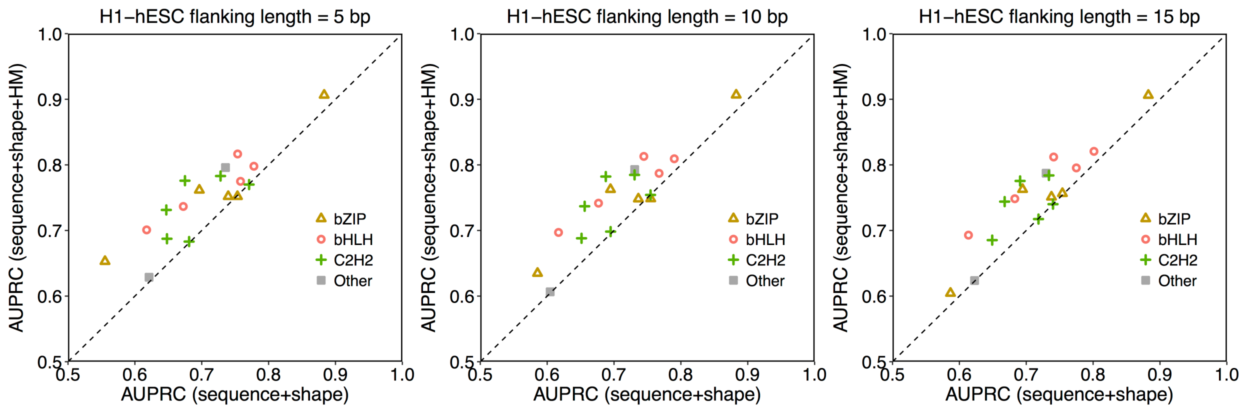

Supplemental Fig. S11. Different length scales of flanking regions in calculating DNA sequence and shape features and their effects on quantitative prediction of in vivo TF binding in the H1-hESC cell line.

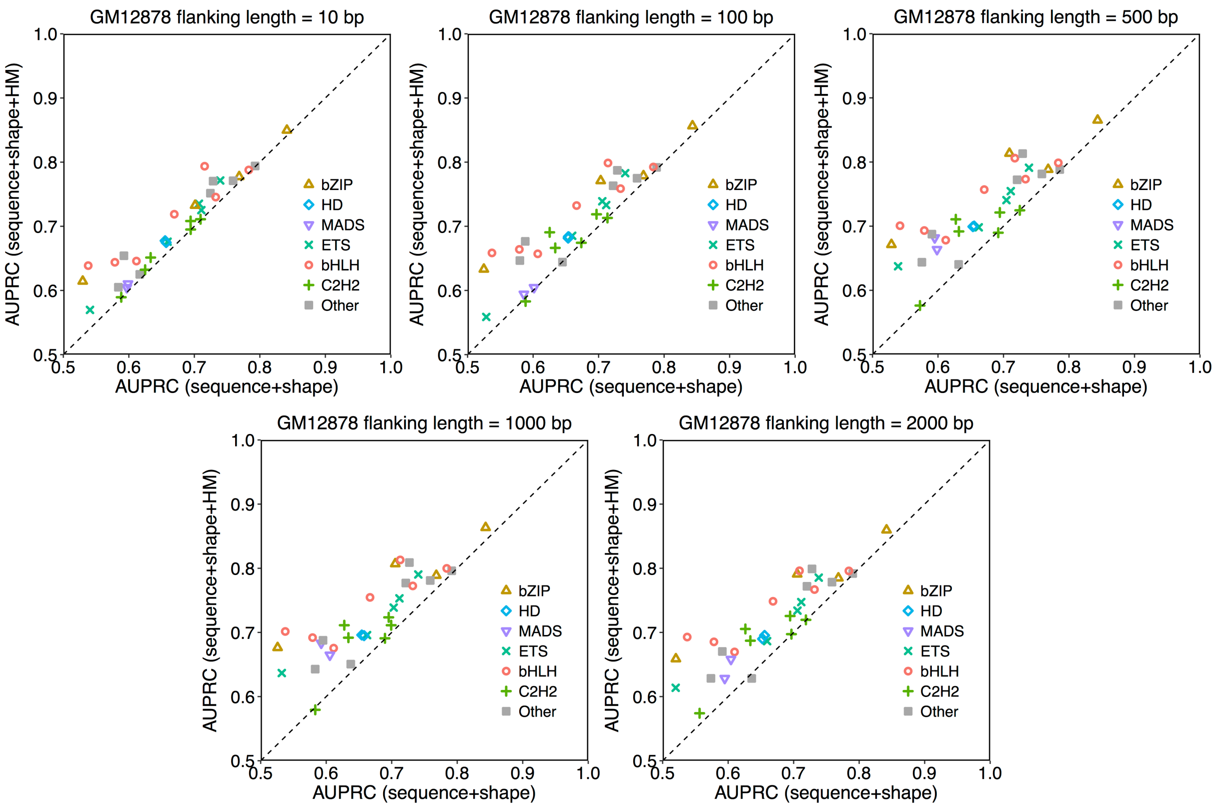

Supplemental Fig. S12. Different length scales of flanking regions in calculating HM patterns and their effects on quantitative prediction of in vivo TF binding in the GM12878 cell line.

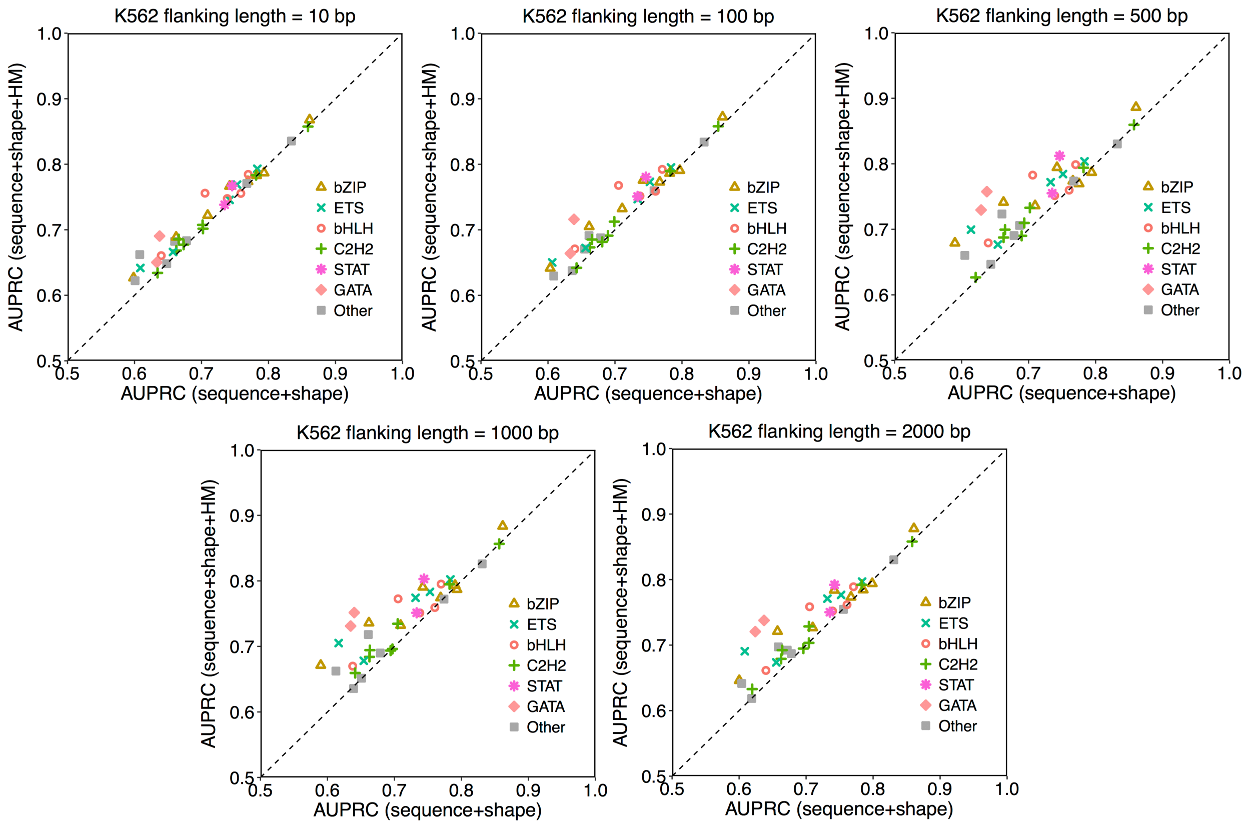

Supplemental Fig. S13. Different length scales of flanking regions in calculating HM patterns and their effects on quantitative prediction of in vivo TF binding in the K562 cell line.

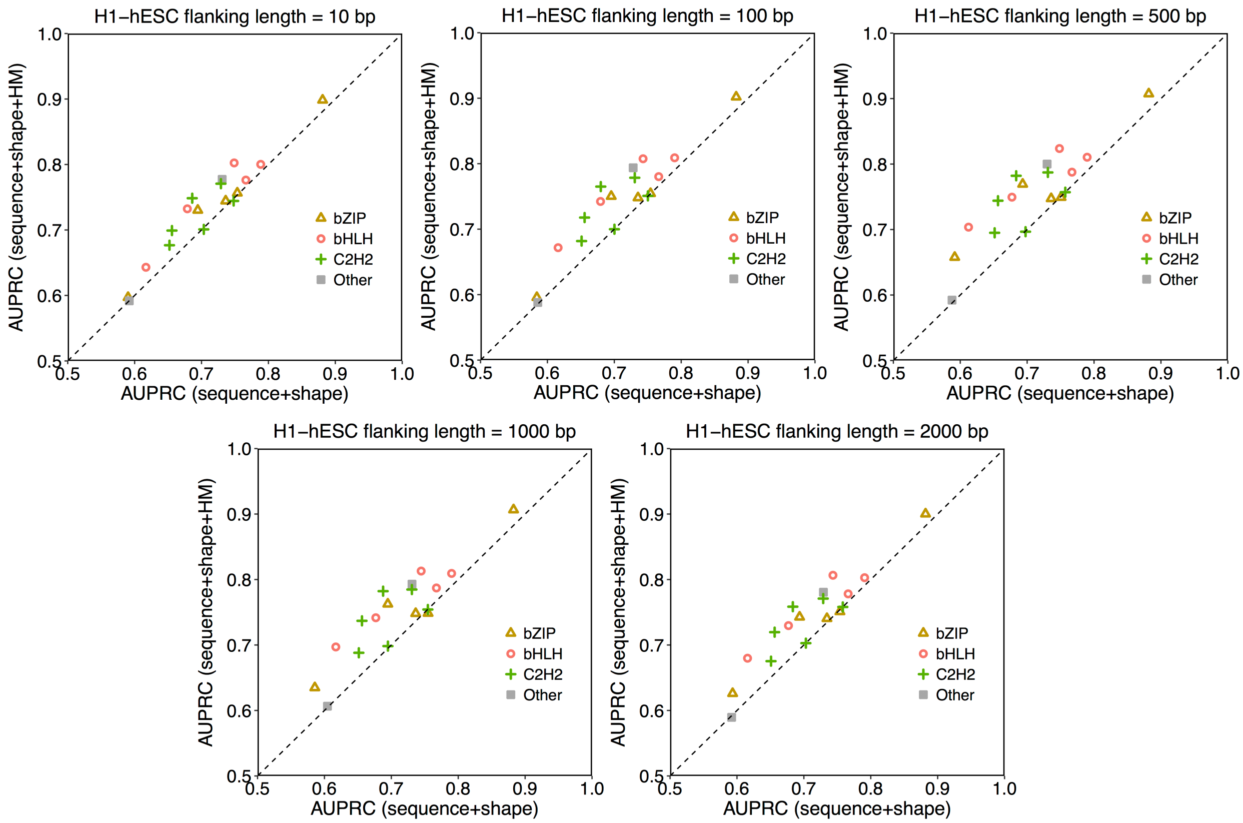

Supplemental Fig. S14. Different length scales of flanking regions in calculating HM patterns and their effects on quantitative prediction of in vivo TF binding in the H1-hESC cell line.

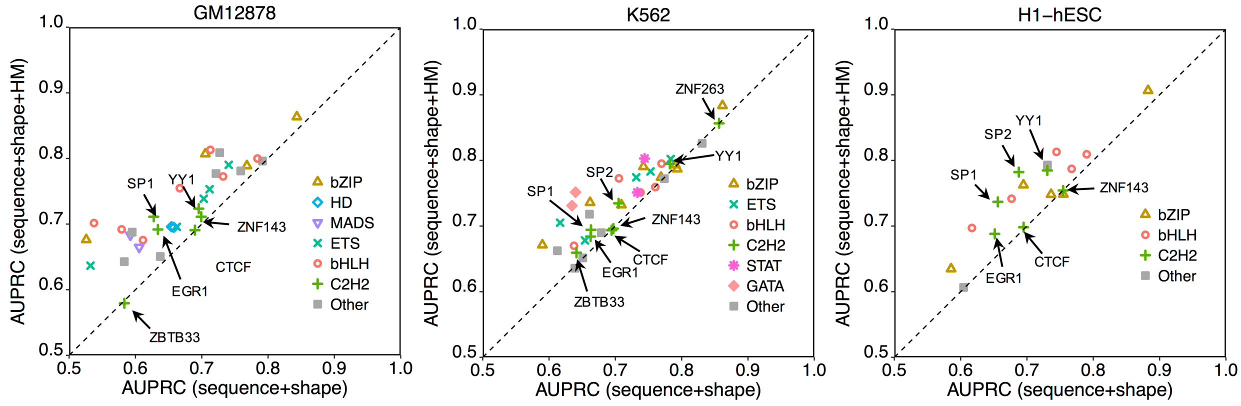

Supplemental Fig. S15. Arrows indicate TFs of C2H2 protein family in Fig. 3.

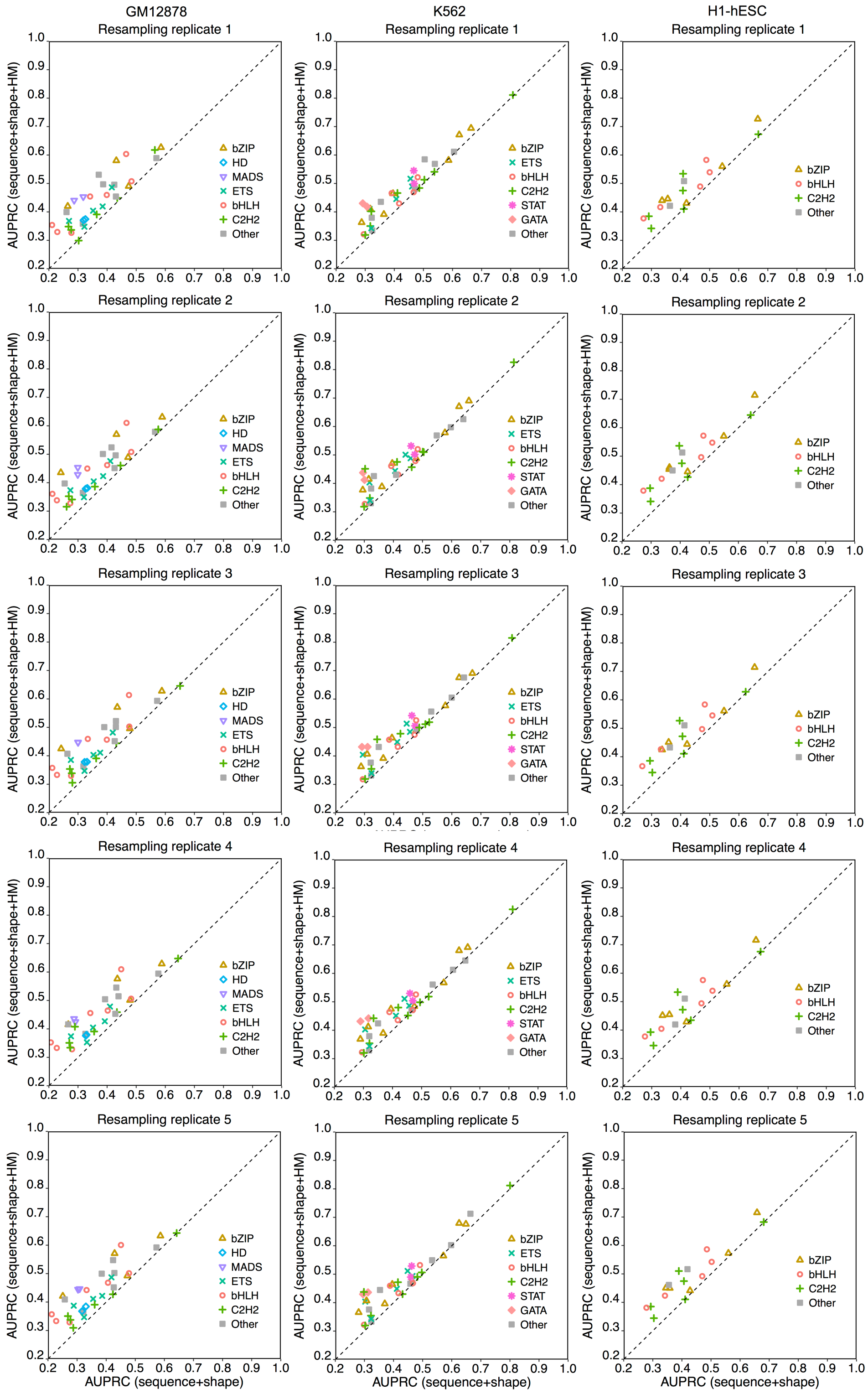

Supplemental Fig. S16. Performance of L2-regularized MLR models in Fig. 3A-C implemented based on resampling of 5x non-BSs compared to non-BSs using bootstrapping with distinct experimental replicates.

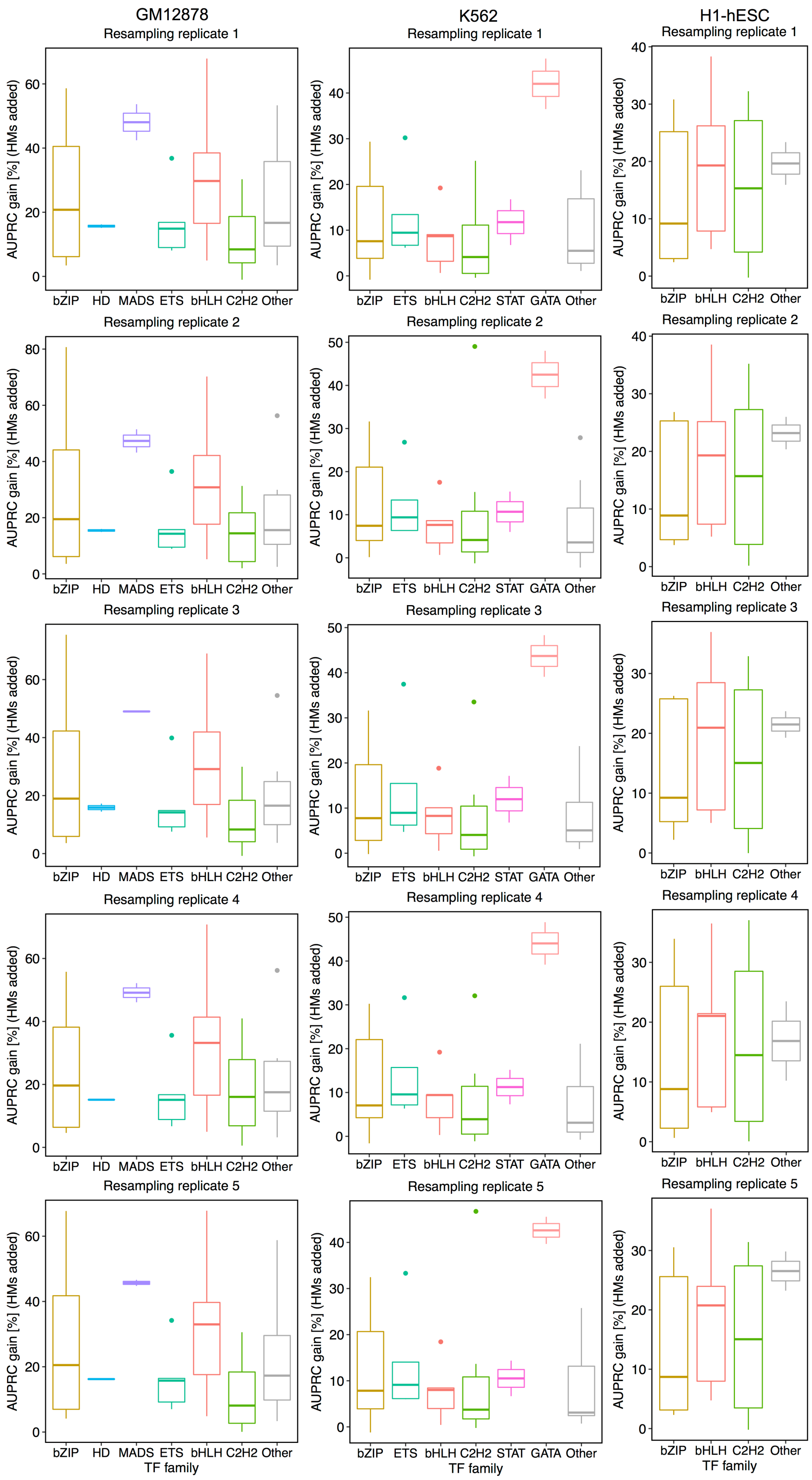

Supplemental Fig. S17. Performance gains in HM-augmented models in Supplemental Fig. S16.

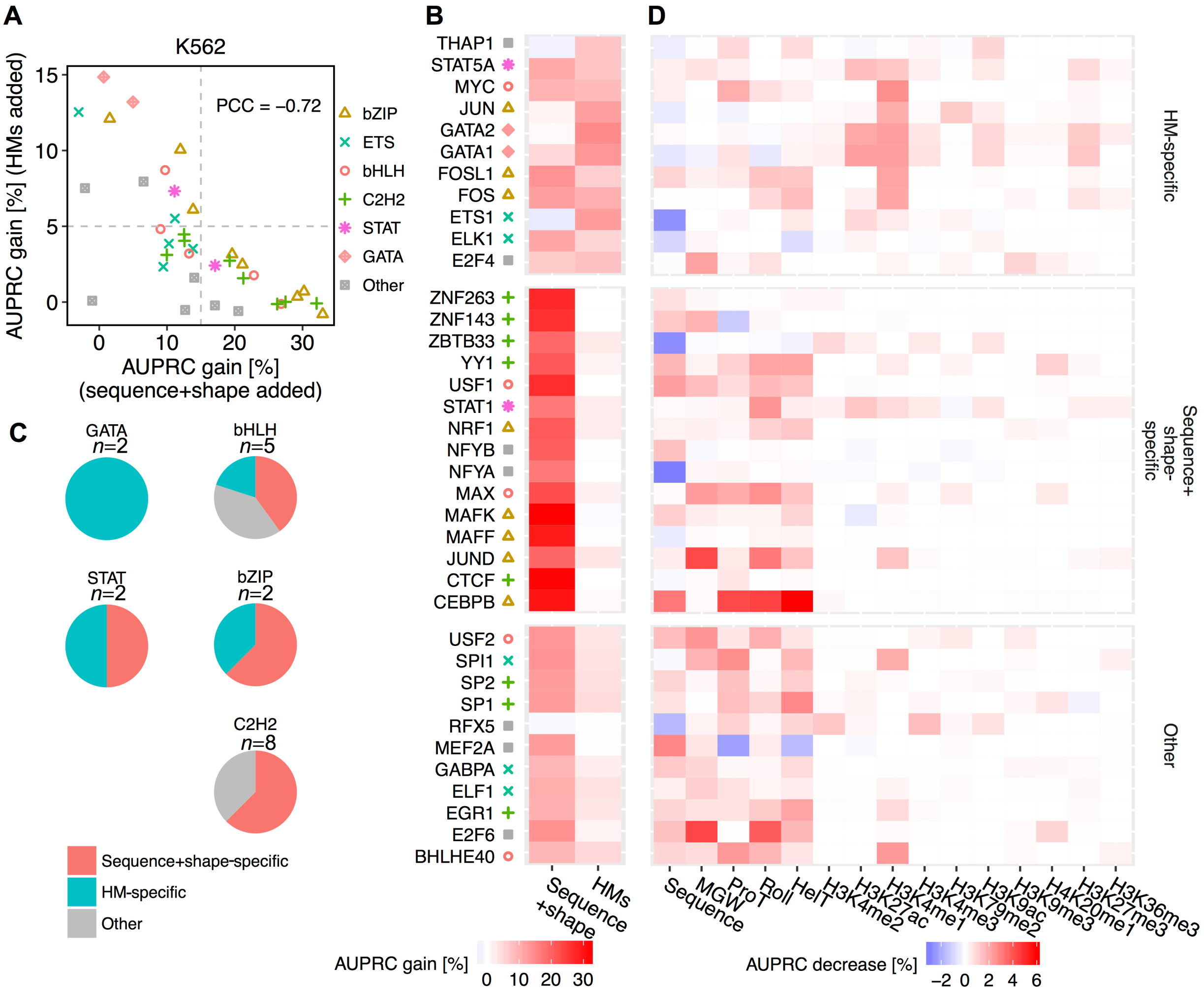

Supplemental Fig. S18. Deconvolution of DNA sequence and shape features at flanking regions of motifs and 10 HM patterns in the K562 cell line. (A) Scatter plot showing performance gain through adding different features. The *x*-axis represents HM pattern-only models as baseline, and recorded performance increase when adding DNA sequence and shape features at flanking regions of motifs. The *y*-axis represents models based on DNA sequence and shape features at flanking regions as baseline, and recorded performance increase through adding HM pattern features. Gray dashed lines intersect with *x*-axis at 15% and with *y*-axis at 5%. The Pearson correlation coefficient (PCC) was calculated between AUPRC gains upon adding these two sets of features. (B) Heat map displaying performance gains when adding either sequence+shape features or HM patterns. With cutoffs shown in gray dashed line in (A), TFs were grouped into sequence+shape-specific, HM-specific, and a group with other features preferred. (C) Pie charts showing the number of TFs with different binding mechanisms in the GATA, STAT, bHLH, bZIP, and C2H2 TF families. (D) Heat map representing the percentage decrease of AUPRC in leave-one-feature-out experiments compared to complete models considering DNA sequence and shape features, and 10 HM features. More intense red color in a cell means larger decrease in performance when leaving out the feature displayed in the *x*-axis for the TF displayed in the *y*-axis.

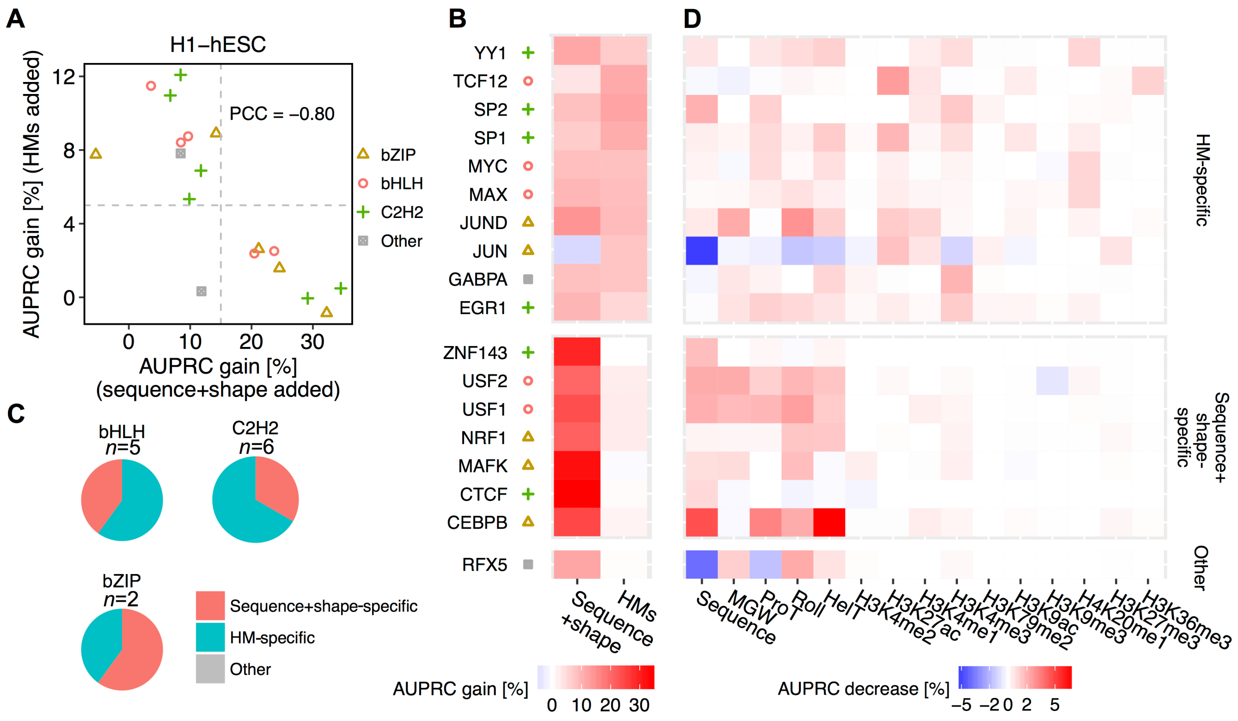

Supplemental Fig. S19. Deconvolution of DNA sequence and shape features at flanking regions of motifs and 10 HM patterns in the H1-hESC cell line. (A) Scatter plot showing performance gain through adding different set of features. The *x*-axis represents HM pattern-only models as baseline, and recorded performance increase when adding DNA sequence and shape features at flanking regions of motifs. The *y*-axis represents models based on DNA sequence and shape features at flanking regions as baseline, and recorded performance increase through adding HM pattern features. Gray dashed lines intersect with *x*-axis at 15% and with *y*-axis at 5%. The Pearson correlation coefficient (PCC) was calculated between AUPRC gains upon adding these two sets of features. (B) Heat map displaying performance gains when adding either sequence+shape features or HM patterns. With cutoffs shown in gray dashed line in (A), TFs were grouped into sequence+shape-specific, HM-specific, and a group with other features preferred. (C) Pie charts showing the number of TFs with different binding mechanisms in the bHLH, bZIP, and C2H2 TF families. (D) Heat map representing the percentage decrease of AUPRC in leave-one-feature-out experiments compared to complete models considering DNA sequence and shape features, and 10 HM features. More intense red color in a cell means greater decrease in performance when leaving out the feature displayed in the *x*-axis for the TF displayed in the *y*-axis.

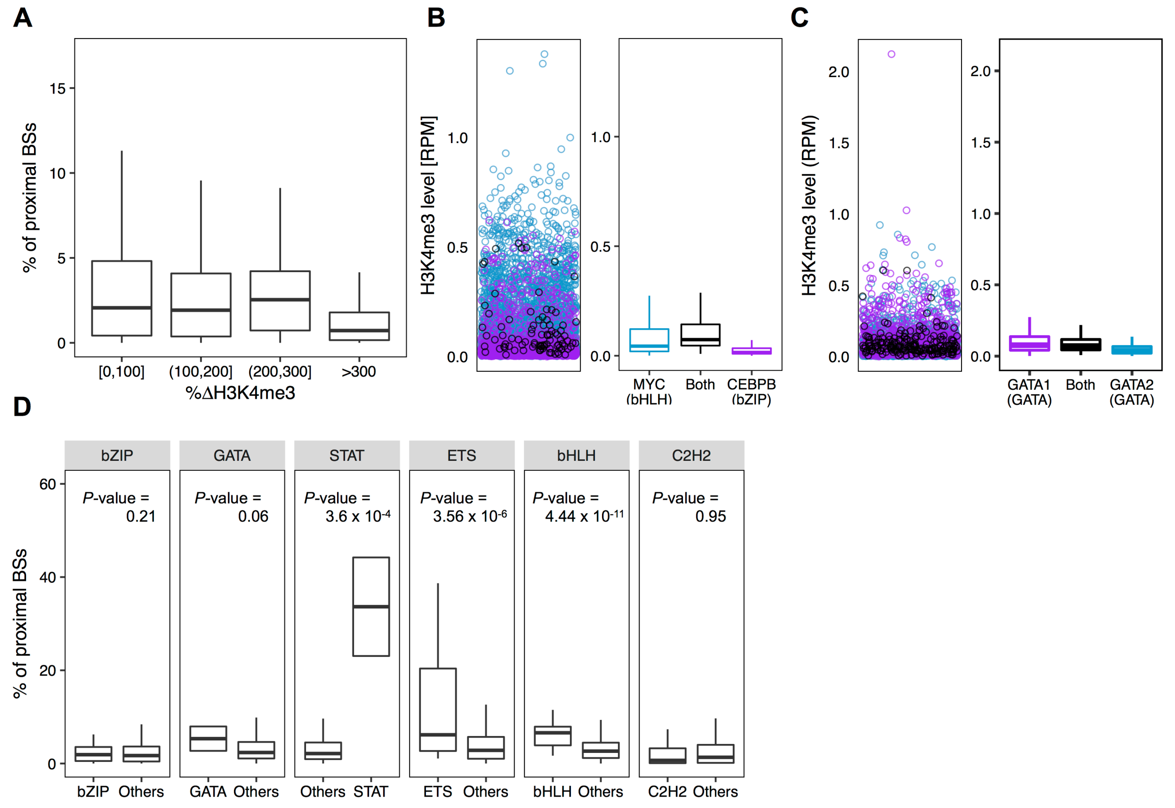

Supplemental Fig. S20. HM environment can constrain TF co-occupancy in the K562 cell line. TFs from the same protein family and TF families with a similarly favorable HM environment (or binding manner) tend to co-localize in the genome. (A) Boxplots of the percentages of BSs of a TF that are in close proximity (within 300 bp) to BSs of each of the other TFs, versus average differences of H3K4me3 surrounding BSs between these two TFs. (B, left) H3K4me3 level surrounding BSs shared (black) by MYC (bHLH TF family) and CEBPB (bZIP TF family), MYC-only (blue), and CEBPB-only (purple). (B, right) Box plots representing the distribution of H3K4me3 level surrounding BSs shared (black) by MYC and CEBPB, MYC-only (blue), and CEBPB-only (purple). (C, left) H3K4me3 level surrounding BSs shared (black) by GATA2 and GATA1 (both from the GATA family), GATA1-only (purple), and GATA2-only (blue). (C, right) Box plots representing the distribution of H3K4me3 level surrounding BSs shared (black) by GATA1 and GATA2, GATA1-only (purple), and GATA2-only (blue). (D) Box plots displaying the distribution of percentages of proximal BSs among intra-family TF pairs and inter-family TF pairs for each protein family. One-sided Wilcoxon test *P*-values show that intra-family TF pairs have significantly higher percentages of proximal BSs compared to inter-family TF pairs.

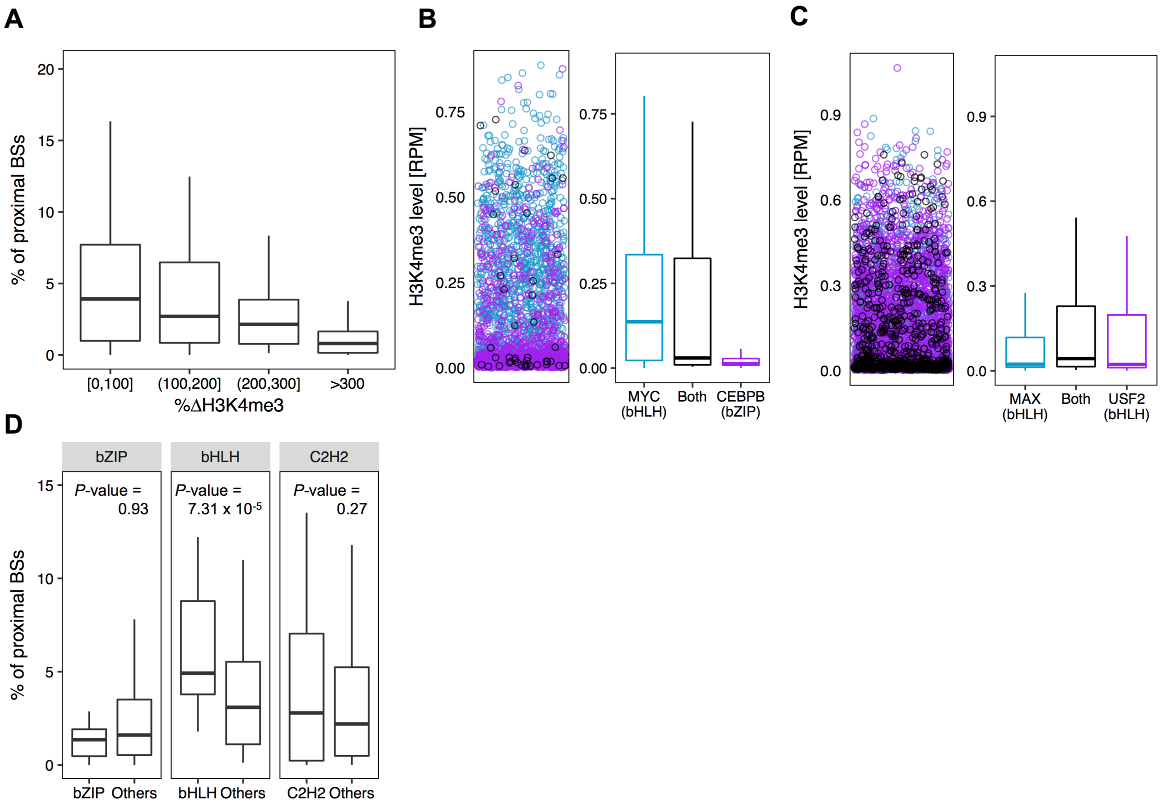

Supplemental Fig. S21. HM environment can constrain TF co-occupancy in the H1-hESC cell line. TFs from the same protein family and TF families with a similarly favorable HM environment (or binding manner) tend to co-localize in the genome. (A) Box plots of the percentages of BSs of a TF that are in close proximity (within 300 bp) to BSs of each of the other TFs, versus average differences of H3K4me3 surrounding BSs between these two TFs. (B, left) H3K4me3 level surrounding BSs shared (black) by MYC (bHLH TF family) and CEBPB (bZIP TF family), MYC-only (blue), and CEBPB-only (purple). (B, right) Box plots representing the distribution of H3K4me3 level surrounding BSs shared (black) by MYC and CEBPB, MYC-only (blue), and CEBPB-only (purple). (C, left) H3K4me3 level surrounding BSs shared (black) by MAX and USF2 (both from the bHLH TF family), USF2-only (purple), and MAX-only (blue). (C, right) Box plots representing the distribution of H3K4me3 level surrounding BSs shared (black) by MAX and USF2, USF2-only (purple), and MAX-only (blue). (D) Box plots displaying the distribution of percentages of proximal BSs among intra-family TF pairs and inter-family TF pairs for each protein family. One-sided Wilcoxon test *P*-values show that intra-family TF pairs have significantly higher percentages of proximal BSs compared to inter-family TF pairs.

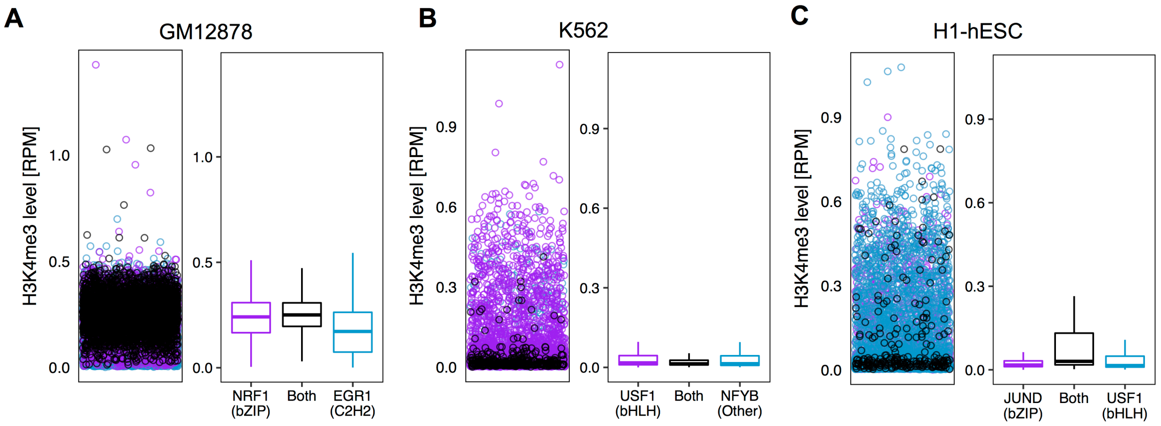

Supplemental Fig. S22. TFs from protein families with a similarly favorable HM environment (or binding mechanism) tend to co-localize in the genome. (A, left) H3K4me3 level surrounding BSs shared (black) by NRF1 (bZIP TF family) and EGR1 (C2H2 TF family), NRF1-only (purple), and EGR1-only (blue). (A, right) Box plot representing the distribution of H3K4me3 level around BSs shared (black) by NRF1 and EGR1, NRF1-only (purple), and EGR1-only (blue) in the GM12878 cell line. (B, left) H3K4me3 level surrounding BSs shared (black) by USF1 (bHLH TF family) and NFYB (NFY TF family), USF1-only (purple), and NFYB-only (blue). (B, right) Box plot representing the distribution of H3K4me3 level around BSs shared (black) by USF1 and NFYB, USF1-only (purple), and NFYB-only (blue) in the K562 cell line. (C, left) H3K4me3 level surrounding BSs shared (black) by JUND (bZIP TF family) and USF1 (bHLH TF family), JUND-only (purple), and USF1-only (blue). (C, right) Box plot representing the distribution of h3k4me3 level around BSs shared (black) by JUND and USF1, JUND-only (purple), and USF1-only (blue) in the H1-hESC cell line.

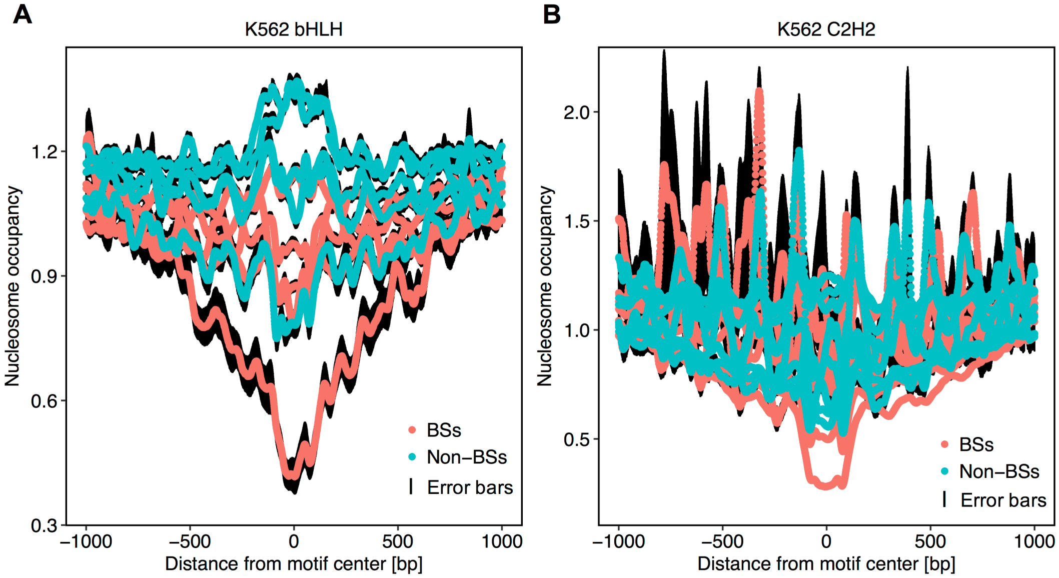

Supplemental Fig. S23. Nucleosome occupancy decreases around BSs compared to non-BSs among TF families that bind in an HM-specific manner. Average nucleosome occupancy in each position 1 kb upstream and downstream around BSs and non-BSs for the (A) bHLH and (B) C2H2 TF families in the K562 cell line. Black edges encompassing the average line represent standard error bars at each nucleotide position.

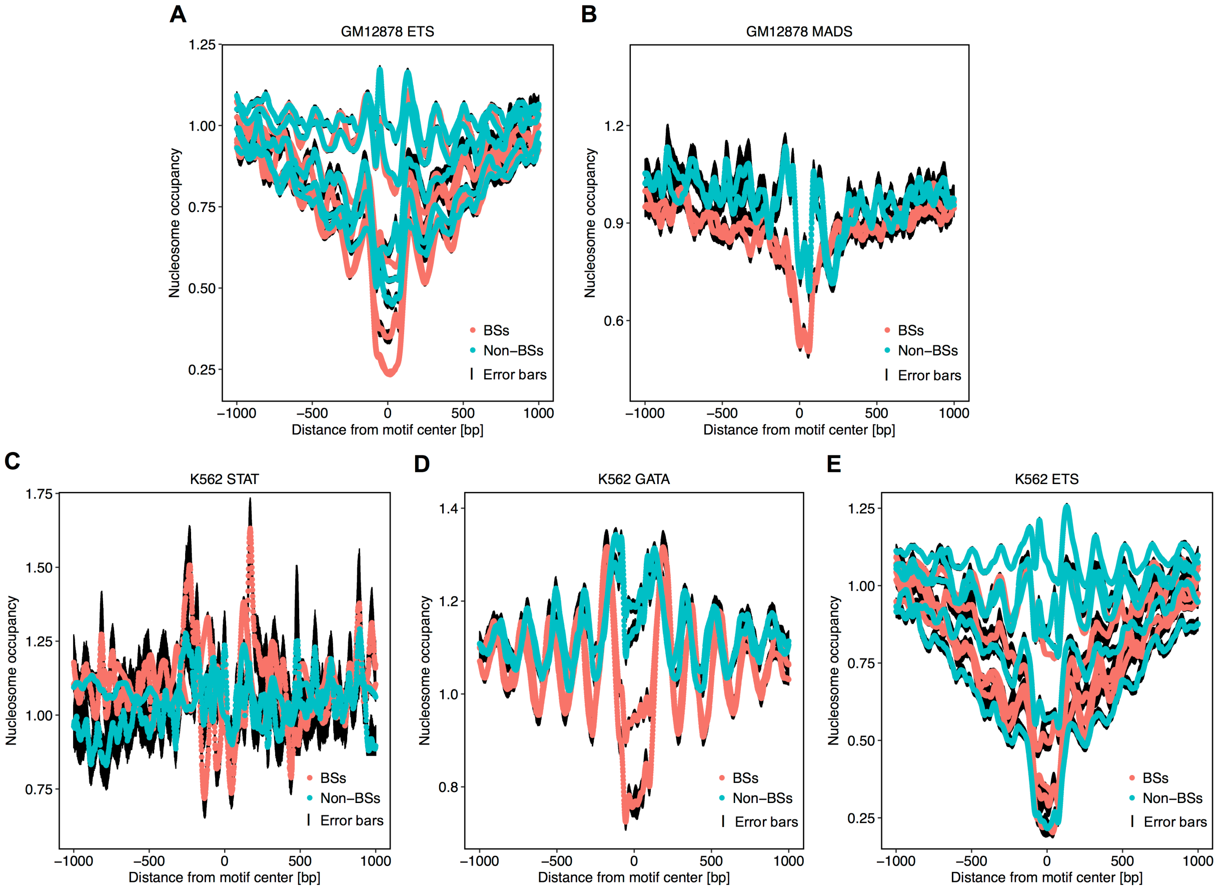

Supplemental Fig. S24. Nucleosome occupancy decreases around BSs compared to non-BSs among TF families that bind in an HM-specific manner. Average nucleosome occupancy in each position 1 kb upstream and downstream around BSs and non-BSs for the (A) ETS and (B) MADS-domain TF families in the GM12878 cell line and for the (C) STAT, (D) GATA, and (E) ETS TF families in the K562 cell line. Black edges encompassing the average line represent standard error bars at each nucleotide position.

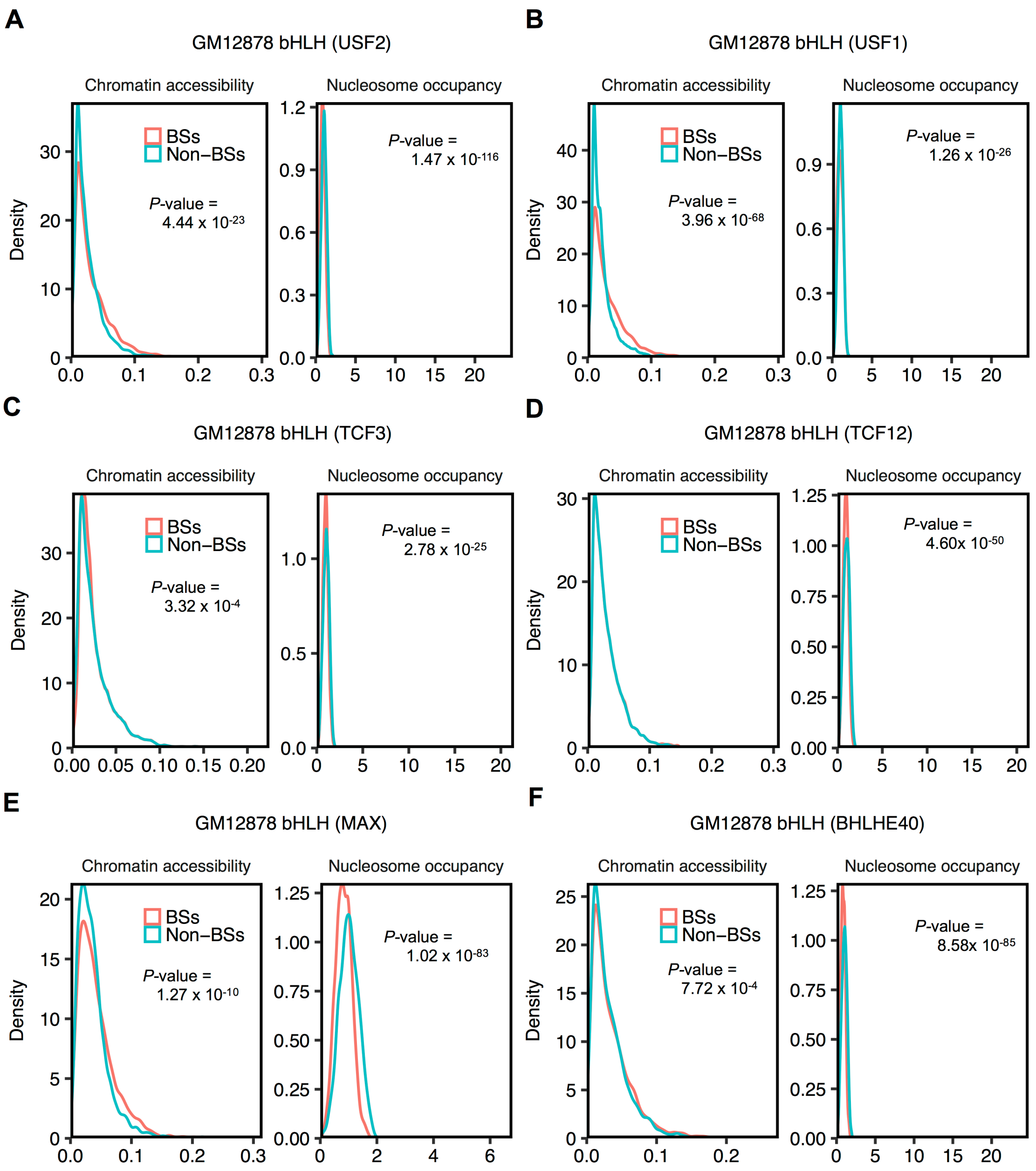

Supplemental Fig. S25. Density plots showing distributions of chromatin accessibility and nucleosome occupancy around BSs and non-BSs for (A) USF2, (B) USF1, (C) TCF3, (D) TCF12, (E) MAX, and (F) BHLHE40 in the bHLH TF family. Two-sided Wilcoxon tests were conducted to test if these distributions have shifts. Only distributions of nucleosome occupancy for BSs exhibiting a significant shift are indicated by the *P*-value (<0.002).

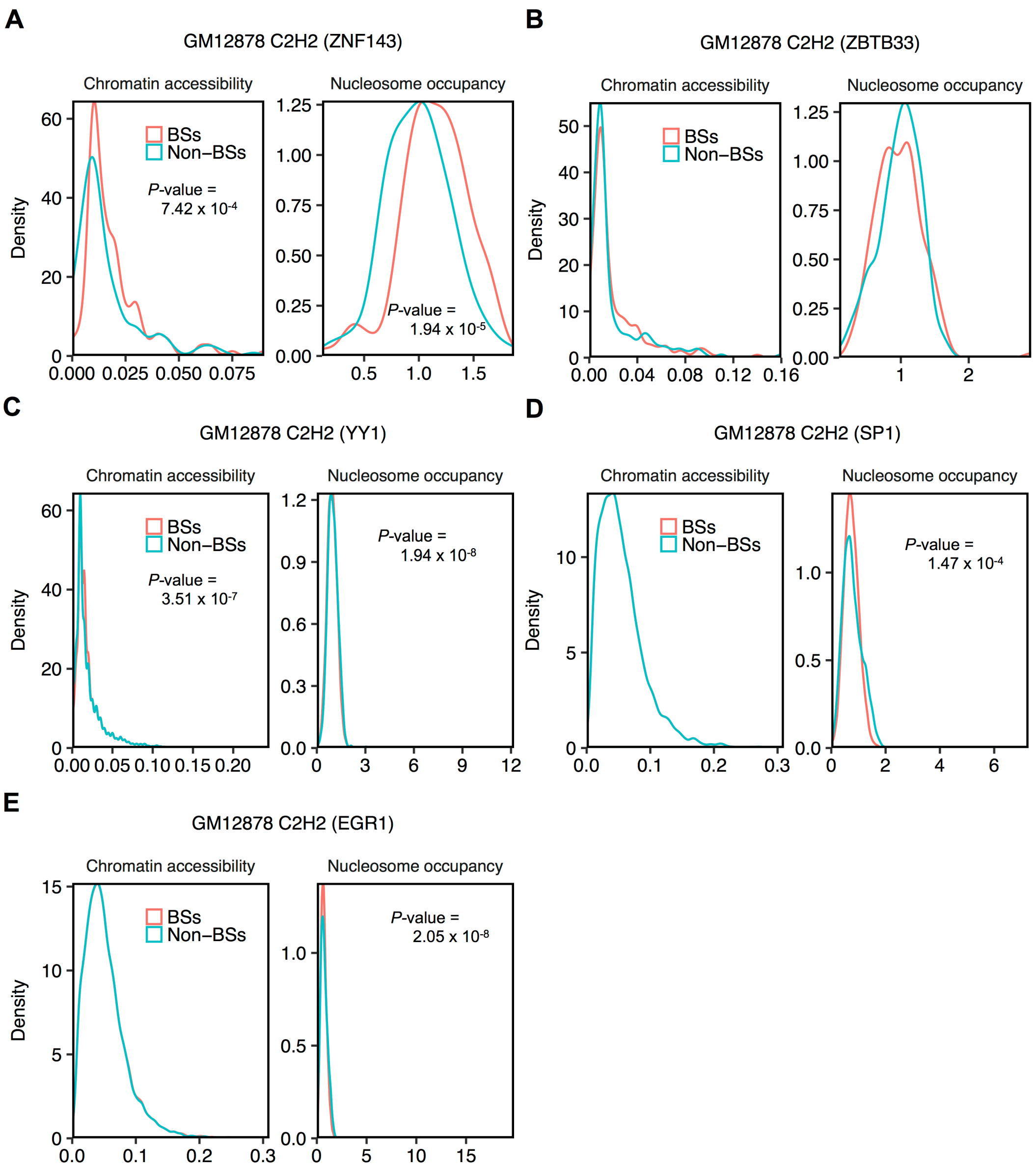

Supplemental Fig. S26. Density plots showing distributions of chromatin accessibility and nucleosome occupancy around BSs and non-BSs for (A) ZNF143, (B) ZBTB33, (C) YY1, (D) SP1, and (E) EGR1 in the C2H2 TF family. Two-sided Wilcoxon tests were conducted to test if these distributions have shifts. Only distributions of nucleosome occupancy for BSs exhibiting a significant shift are indicated by the *P*-value (<0.002).

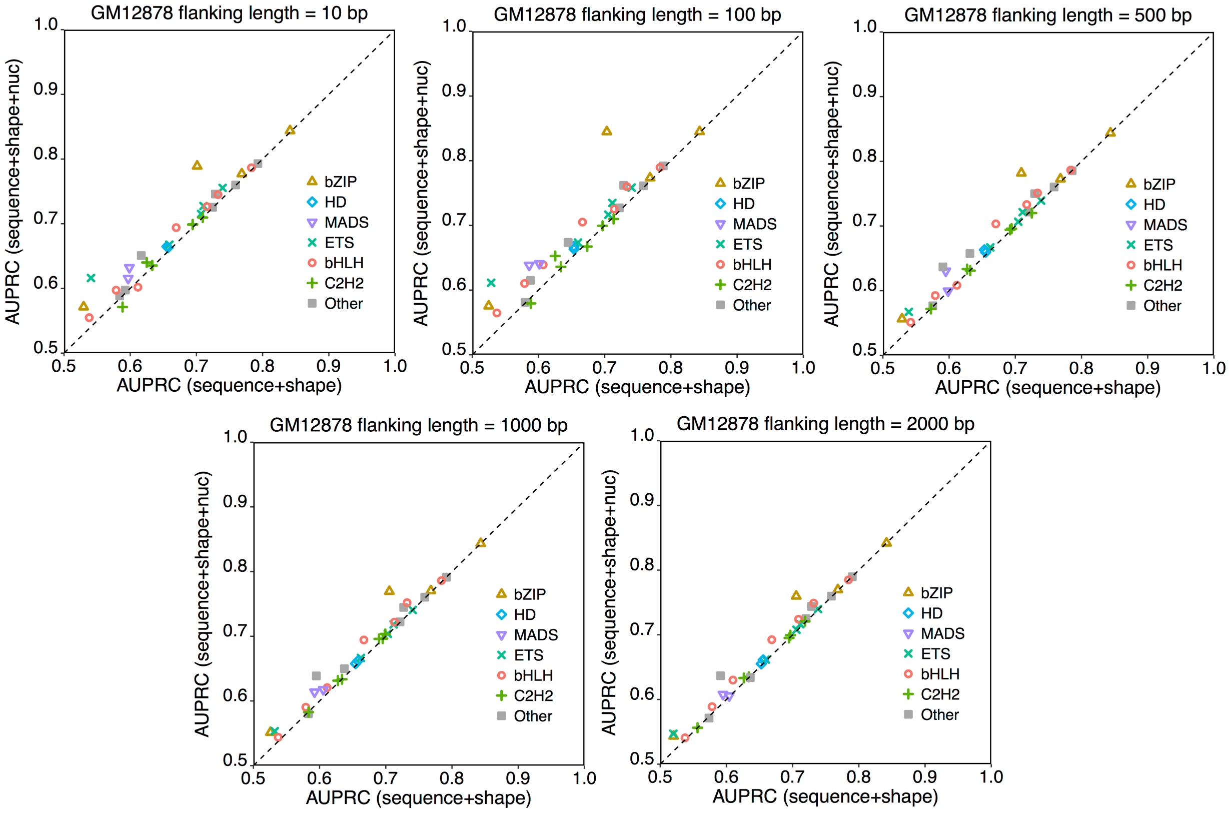

Supplemental Fig. S27. Different length scales of flanking regions in calculating nucleosome occupancy and their effects on quantitative prediction of in vivo TF binding in the GM12878 cell line.

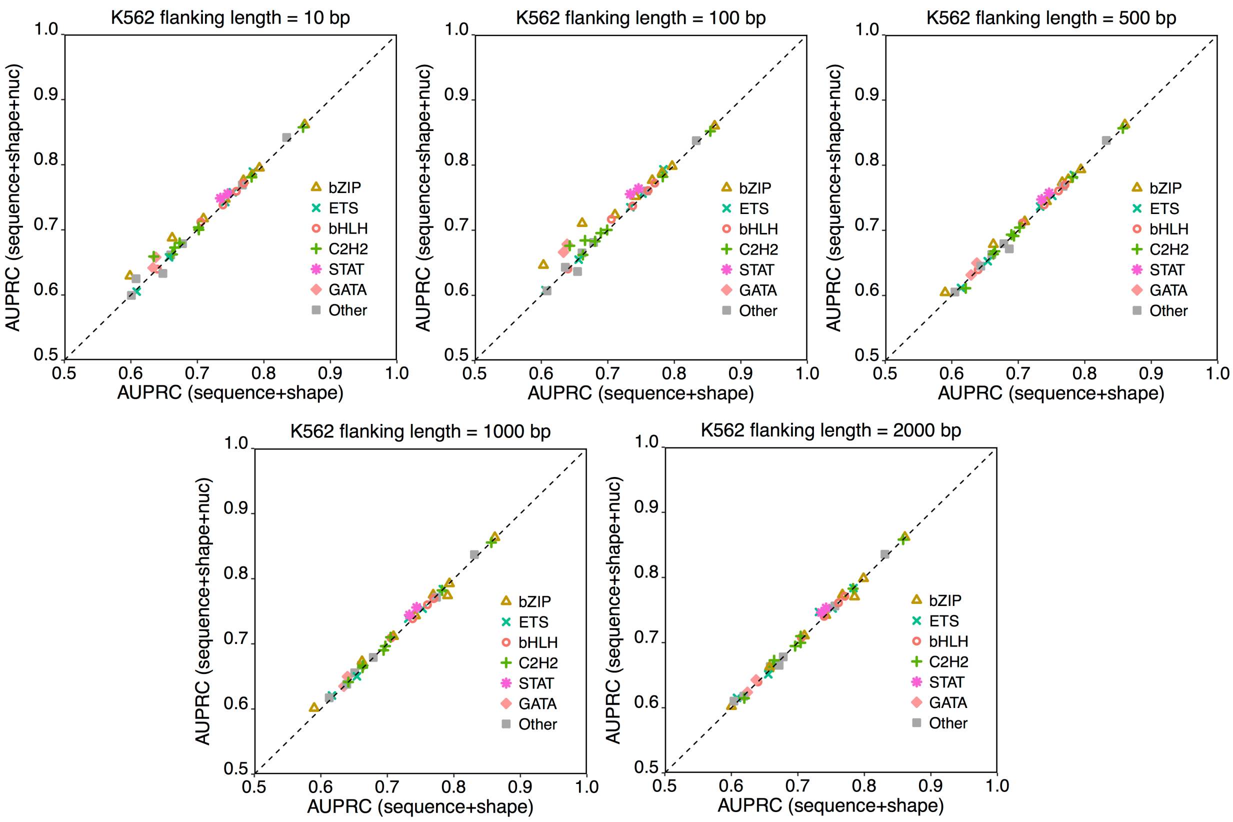

Supplemental Fig. S28. Different length scales of flanking regions in calculating nucleosome occupancy and their effects on quantitative prediction of in vivo TF binding in the K562 cell line.

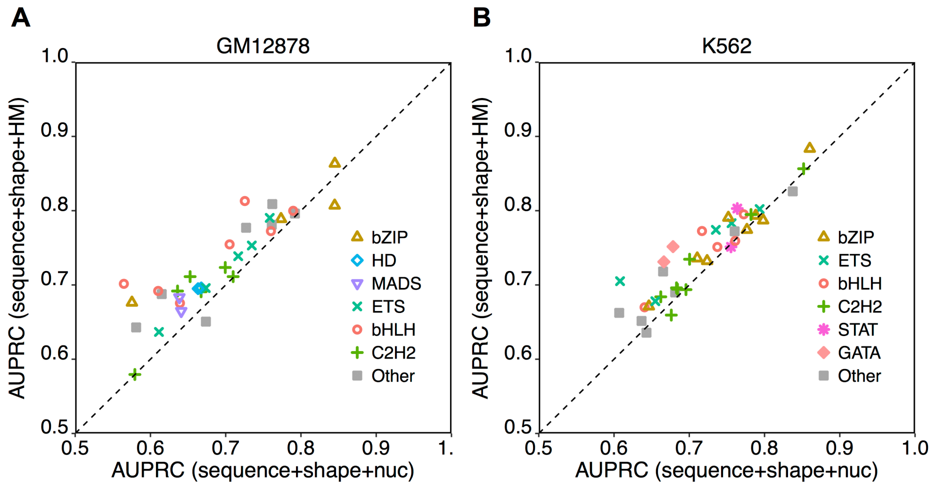

Supplemental Fig. S29. Sequence+shape+HM models outperform sequence+shape+nuc models in the (A) GM12878 and (B) K562 cell lines. We choose sequence+shape+nuc models with the 100-bp motif environment and choose sequence+shape+HM models with the 1-kb motif environment because these lengths achieved the best performance.
