# Supplementary figures and images for: Relationship between histone modifications and transcription factor binding is protein family specific
Source: Genome Res. 2018 Mar;28(3):321–33. doi: 10.1101/gr.220079.116 (PMC5848611; doi:10.1101/gr.220079.116)

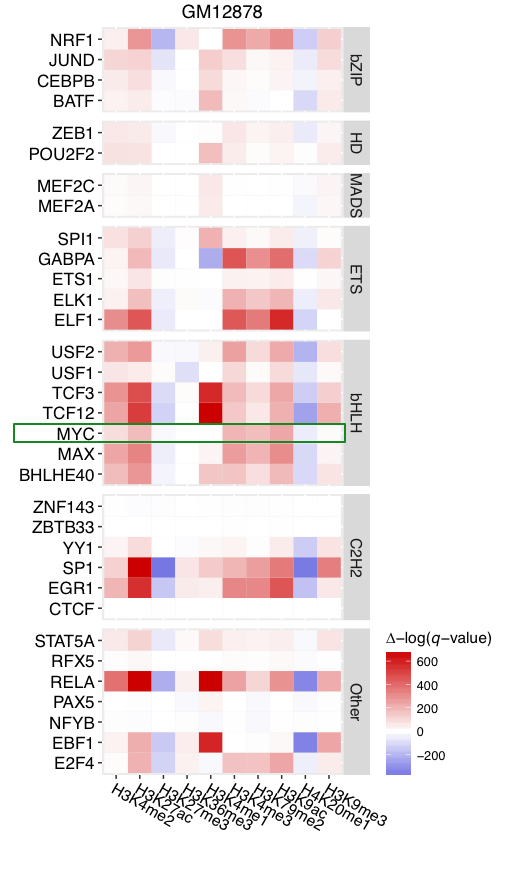

Supplement: Supplemental Material [file supp_gr.220079.116_Supplemental_Material.zip › Supplemental_Material/MYC/Figure_2A_MYC.png]

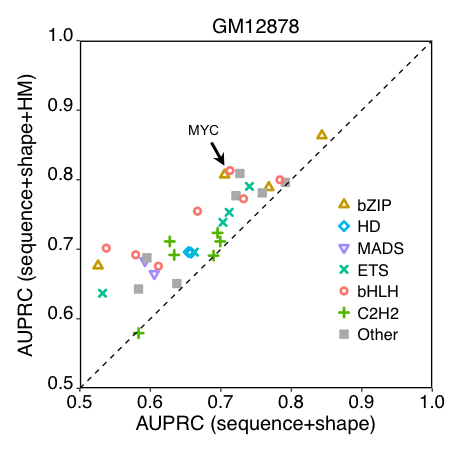

Supplement: Supplemental Material [file supp_gr.220079.116_Supplemental_Material.zip › Supplemental_Material/MYC/Figure_3A_MYC.png]
